# Approach to the Chemotaxonomic Characterization of Traditional Cultivation Grape Varieties through Their Varietal Aroma Profile

**DOI:** 10.3390/foods11101427

**Published:** 2022-05-15

**Authors:** Ángela Díaz-Fernández, Emilia Díaz-Losada, Sandra Cortés-Diéguez

**Affiliations:** 1 Estación de Viticultura e Enoloxía de Galicia-AGACAL, Ponte San Clodio s/n, 32428 Ourense, Spain; angela.diaz.fernandez@xunta.es (Á.D.-F.); emilia.diaz.losada@xunta.es (E.D.-L.); 2 Edificio Campus Auga, Biotecnología Industrial e Ingeniería Ambiental, BiotecnIA, Campus Sur, Universidad de Vigo, 32004 Ourense, Spain

**Keywords:** *Vitis vinifera* L., varietal diversity, varietal characterization, volatile compounds, aroma profile, chemotaxonomic markers, fingerprint

## Abstract

In this study, the aroma profile of 12 minority grape varieties of *Vitis vinifera* L., included in the ‘Caiño group’, was defined along three vintages by solid phase microextraction followed by the gas chromatography–mass spectrometry method (SPME-GC-MS). Principal objectives were to assess the aromatic profile as a useful fingerprint to differentiate them, recover traditionally cultivated grape varieties for the differentiation of an important wine-growing area and discover their chemotaxonomic potential. In each variety, free and bound volatile profile was carried out by grouping varietal compounds into thirteen families. In total, 339 volatile compounds were identified, 230 as free forms and 205 as aromatic precursors. Remarkable quantitative differences were observed between aromatic profiles for terpenes in the free fraction and for C_6_ compounds, alcohols, sesquiterpenes and phenols in the glycosidic fraction. Principal component analysis based on their aromatic profile highlights a good differentiation between varieties and suggests a certain degree of aromatic chemotaxonomic proximity between previously known parental varieties, ‘Caiño Blanco’ with respect to ‘Caiño Bravo’ and ‘Albariño’. This study shows the preliminary results of a large research project involving a larger number of grape varieties and thus a broader spectrum of genetic relationships between them.

## 1. Introduction

The impoverishment of grapevine genetic pool is the result of a homogenization process in the international wine trade. The few number of varieties included in quality schemes such as Protected Designation of Origin (PDO) or Protected Geographical Indication (PGI), which cover a large proportion of the grapevine surface, has led to a big loss of genetic diversity [1,2,3].

These days, enhancing the recovery and promotion of grapevine genetic richness is of the utmost importance. Among the different reasons for this demand, it could be mentioned that the requirement of improving the grapevine crop towards a greater environmental sustainability, as well as an increase in the differentiation and typicity of the wines from different regions, is the demand of several wine consumers nowadays [4].

Currently, at least 6000 *Vitis vinifera* L. varieties are grown across the world for research or production [5]. From known grapevine varieties, 13 of them cover more than the third part of the worldwide vineyard, and 33 of them cover 50% of the vineyard surface. Some varieties are known as ‘international varieties’ for their broad distribution, while other varieties may be widely cultivated but only in a few countries [6]. Several factors such as climatic change, the urgent reduction of phytosanitary inputs into the field, search for alternatives to the conventional fight against emerging vineyard diseases and increasing the offer of different wines that fulfil the wine consumers demands, involve studying and recovering minority varieties, almost extinct, in different wine traditional regions [7]. The broad genotypic variation among existing varieties implies high variation of traits. The possibility of using different or even new genotypes would be potentially powerful means of adaptation to the previously mentioned challenges. Among other characteristics, those varieties with higher tartaric/malic ratio could be better adapted to warmer conditions [8], one of the main consequences of climate change. Despite that, big homogenization of varieties across the globe, some Old World regions have maintained a higher diversity of varieties. Existing grapevine germplasm banks all around the world, established to conserve endangered native varieties, could be used nowadays as an interesting resource to promote and diversify scarce diversity producing vineyards from existing genetic material. It is considered of great importance to carry out a deep study of different traits across experimental grapevine germplasm banks located in different climatic regions. This could be useful to better understand the behavior of different varieties and the degree of influence that genetics and the environment has on their different traits [9], given that, to increase resistance to climate change, diversity must agree with those most necessary traits to adapt to future climate scenarios [10].

Nowadays, there is a trend to implement numerous studies of those minority or autochthonous grapevine varieties [3], because despite the fact that wine growers have selected different clones from several varieties for their vineyards along years, the diversity at the variety level implies a greater phenotypic spectrum [9]. There have been several studies of characterization of minority varieties across the world in this last decade, Loureiro et al. [7] with the agronomical characterization of six minority varieties from Asturias (North Spain), Balda et al. [11] characterized agronomic and oenologically 16 minority varieties from D.O. Ca Rioja and García-Muñoz et al. [2], as well as López et al. [12], who carried out the aromatic characterization of 21 and 78 minor varieties, respectively. Phenolic characterization of different minority varieties has been developed by Alcalde-Eón et al. [13] with the ‘Rufete’ variety as well as by Álvarez-Casas et al. [14] with 6 autochthonous white varieties from the northwestern Iberian Peninsula and by Costa et al. [15], in which they characterized the anthocyanin profile and antioxidant activity of 24 varieties cultivated in two Portuguese regions under those specific environmental conditions.

In the northwest of the Iberian Peninsula, is located the experimental grapevine germplasm bank of Estación de Viticultura y Enología de Galicia (EVEGA), founded in the 1980s with the main objective of conserve minority and almost extinct traditionally cultivated varieties from this traditional wine-growing area [1]. Previous genetic studies carried out in EVEGA with SSRs molecular markers revealed similarities among those varieties conserved in the germplasm bank which have allowed us to discover the population structure, phylogeny and geographic origin of conserved varieties [1,16,17,18]. One of those population groups, the reconstructed population 1 group a (RPP1a), which includes most of the Galician varieties of the germplasm bank, embraces the Caiño group. This is considered the most ancient lineage of grapevine in the northwestern area of the Iberian Peninsula. Its origin variety is considered ‘Caíño Bravo’ synonym of ‘Amaral’ (‘Azal Tinto’) in Portugal [19] given the high number of alleles that it shares with other varieties included in the group such as ‘Castañal’, ‘Sousón’, ‘Caíño Longo’, ‘Caíño Tinto’ (syn. ‘Caiño Redondo 1’) and ‘Caíño Blanco’ [16,20]. It even has a parental relationship with some of them, such as ‘Caíño Blanco’, probably descended from ’Caiño Bravo’ and ‘Albariño’ [17]. Some other minority varieties could also be included in the Caiño group despite not having such a close relationship with ‘Caiño Bravo’. Those are: ‘Brancellao’, ‘Loureira’, ‘Albariño’ and ‘Verdello Blanco’ [17]. Generally, all varieties included in the Caiño group are characterized by their lower potential alcohol degree, longer vegetative cycle and higher acidity rate.

In addition to the genetic data previously mentioned, recently Cunha et al. [21] have found a possible relationship between *Vitis vinifera* L. cultivated varieties grown in the north of Portugal, close to the Galician area, and *Vitis vinifera* L. *sylvestris*. This study brings out a possible close genetic relationship between ‘Caiño Bravo’ (syn. ‘Amaral’) and *V. vinifera* L. *sylvestris* and would support other phenotypic and physicochemical similarities among ‘Caiño Bravo’ grapes with those of *V. vinifera* L. *sylvestris*. Both show small bunches, dark blue grapes and high acidity rates. Furthermore, ‘Bravo’ means sylvestris in Galicia.

In addition to these interesting genetic research findings, there is an oenological known quality of some of the varieties of the group. ‘Albariño’ and ‘Loureira’ in Spain (synonyms of ‘Alvarinho’ and ‘Loureiro’, respectively in Portugal) and ‘Caiño Blanco’ in Portugal are the most important varieties for the elaboration of monovarietal white wines [17] in the northwestern area of the Iberian Peninsula, including Galicia and regions from the North of Portugal. With respect to the red varieties, the climate change incidence makes it necessary to have varieties with lower potential alcohol degree, higher levels of acidity and high polyphenolic content, all of them general characteristics of Caiño group red varieties [21].

In addition to this oenological potential based on their physicochemical parameters and due to the importance of the volatile composition of a wine in its quality [22], the study of the aromatic composition of these minority varieties is also very important to complete their oenological characterization. Aromatic compounds of grapes such as monoterpenes, C_13_-norisoprenoids, methoxypyrazines, C_6_ alcohols, benzene or sulphur compounds, which would be considered the potential varietal aroma of the wines, have a very important role in the quality as well as in the typicity of wines [23,24,25]. In addition to their importance in wine quality, the different aromatic compounds in grapes have also been evaluated to serve as varietal markers, useful for varietal differentiation. The varietal aroma compounds are different for each grape variety, they have been considered for grapevine varietal characterization and classification [26] as they impart specific aromas to each wine [27]. Marais J. [28] already related terpenes and other compounds with terpenic origin, as important grape varietal compounds that characterized aromatic wines, not only from muscat but also from non-muscat cultivars. C_6_ alcohols have been assessed as varietal markers for the identification of the origin of the wine [24]. The variation in key molecules from different families (terpenes, alcohols, or aldehydes) as well as the identification of specific aromatic descriptors have been also used as markers for specific varieties [22]. However, other groups of volatile compounds must be considered for the role they could also play in wines; for example, aldehydes, such as hexanal or (*E*)-2-hexenal, different alcohol compounds, such as aromatic alcohols as benzyl alcohol or alcohols from 4 to 11 carbon atoms, or ketones [29].

Considering the above arguments, the aims of this study were to (1) identify the aromatic compounds of 12 grape varieties genetically included in the Caiño group, (2) evaluate if these aromatic compounds can classify or group them, (3) compare this classification with the one based on molecular markers and finally (4) evaluate if this characterization could be used as a chemotaxonomic tool.

## 2. Materials and Methods

### 2.1. Plant Material

Grapes from 12 genotypes of Vitis vinifera L. from EVEGA germplasm bank: ‘Albariño’, ‘Brancellao’, ‘Brancellao Blanco’, ‘Caiño Blanco’, ‘Caiño Bravo’, ‘Caiño Longo 1’, ‘Caiño Longo 2’, ‘Caiño Tinto’, ‘Castañal’, ‘Loureira’, ‘Sousón’ and ‘Verdello Blanco’ were analyzed in 2015, 2016 and 2017. The experimental vineyard is located in Ourense, (Galicia, Spain), northwest of the Iberian Peninsula (42°21′34.5″ N, 8°07′08.2″ W, elevation 87 MAMSL).

Vines were grafted on 196-17C rootstock. Spacing 1.8 m and 1.2 m between rows and within the row, respectively. Rows were oriented in the east–west direction and vines were trained into vertical trellis and formed in espalier using a simple Cordon Royat pruning system, with an approximate age of 30 years old. Cultivars were in duplicate plots of 6 to 11 vines and all plants had received identical protection treatments and were subjected to identical cultivation practices.

Grapes were manually harvested over three consecutive vintages (2015–2017), setting the date according to the sugar content, pH, acidity and sanitary conditions, monitoring the ripening process weekly from the early stages of ripening.

Approximately 500 grapes were randomly collected from each variety, picked from different parts of the bunch (top, central, and bottom), also taking into consideration the number of berries per bunch and the shadow and sun exposure of them. Samples were collected in plastic bags and transported from the experimental vineyard with ice in a small thermal bag. Then, at the laboratory, two samples of 100 grapes were frozen at −20 °C until extraction and determination of varietal volatile compounds and two aliquots of 100 grapes were randomly selected for analysis of the must oenological parameters.

### 2.2. Climatic Parameters

Weather conditions were measured with an automated meteorological station located in the vineyard (iMETOS, Pessl Instruments GmbH, Weiz, Austria). Climatic indices were calculated, in relation with the water balance and thermal conditions: Heliothermal index (HI) corresponding with Huglin index [30] and cool night index (CI). Minimum, maximum, and mean temperatures, rainfall and days with temperatures above 35 °C were estimated for the vegetative (1 April–30 September) and for the annual period (Table S1).

### 2.3. Oenological Parameters

Total soluble solids (TSS, ºBrix), pH and titratable acidity (g tartaric acid·L^−1^) were determined by Fourier transform infrared spectrometry (FTIR) (OENOFOSS™, FOSS, Hilleroed, Denmark). Malic acid and tartaric acid (g·L^−1^) were determined using a chemical autoanalyzer (LISA 2000, HYCEL DIAGNOSTICS, Massy, France, calibrated according to the official methods [31]. In addition, two maturation indices were estimated: De Cillis and Odifredi (MI-CO), that relates sugar (g·100 mL^−1^) and total acidity (g·L^−1^), and Baragiola and Scuppli (MI-BS) in which the relationship between tartaric and total acidity (g tartaric acid·L^−1^) is calculated.

### 2.4. Volatile Compounds

#### 2.4.1. Standards and Reagents

Reference compounds and internal standards (3-octanol and 4-methyl-2-pentanol) were purchased from Sigma-Aldrich (Steinheim, Germany). Absolute ethanol, dichloromethane and methanol, used as solvents, were of high-performance liquid chromatography (HPLC) gradient grade and were purchased from Merck (Darmstadt, Germany). Pure water was obtained from a Mili-Q purification system (Milipore, Bedford, MA, USA).

##### Free Volatile Compounds

The grape free volatile compounds were extracted using solid phase microextraction (SPME) adapting the methodology reported by Perestrelo et al. [32]. The fiber used in the analyses was a Divinylbenzene/Carboxen/Polydimethylsiloxane (DVB/CAR/PDMS) of 2 cm 50/30-μm (Supelco, Bellefonte, PA, USA). Prior to use and according to the manufacturer’s recommendations, the fiber was conditioned at 270 °C for 60 min in the GC injector.

Frozen grape samples, previously destemmed, were grounded using an Ultraturrax and then immediately centrifuged at 6000 rpm for 15 min. A clear juice was obtained for analysis.

In total, 5 mL of the clear juice, 20 µL of 3-octanol (1 g·L^−1^ in ethanol) and 20 µL of 4 methyl-2-pentanol (10 mg·L^−1^ in ethanol) as internal standards, 1.5 g of NaCl and a magnetic stirrer were placed into a 10 mL vial with a polypropylene cap with hole and a PTFE/silicone septum. Each vial was equilibrated for 2 min at 60 °C and 500 rpm in a water bath placed on a heating platform agitation. After the equilibration time, the conditioned fiber was exposed to the sample headspace for 25 min at 60 °C and 500 rpm. After sampling, the desorption of analytes from the fiber coating was made in the injection port of the gas chromatograph at 250 °C for 5 min in splitless mode.

##### Glicosidically Bound Volatile Compounds

C-18 reserve phase solid phase extraction (SPE) was applied for the extraction of precursor volatile compounds as previously reported by Di Stefano et al. [33], later adapted by Diéguez et al. [34]. Volatile compounds were separated on 1 g C-18 cartridges (Hypersep Spe 1000 mg C-18, Thermo scientific, Waltham, MA, USA), previously conditioned with 5 mL of methanol and 10 mL of water. Twenty-five mL of the clear juice diluted 1:1 with distilled water passed through the column. Free volatiles were eluted with 10 mL of dichloromethane. Afterwards, the cartridge was washed with 25 mL of distilled water to elute the water-soluble compounds, and finally, the aroma precursors were eluted with 10 mL of methanol. Eluted methanol was evaporated to dryness using a vacuum (Rotovapor^®^ R-215, BUCHI, Flawil, Switzerland) and then reconstituted in 5 mL of citrate-phosphate-buffered solution (pH 5.0). In total, 200 μL of the AR 2000 (Rapidase, DSM food specialties, Seclin, France) solution (100 g·L^−1^ in 0.1 mol·L^−1^ citrate-phosphate buffer, pH 5.0) was added and vortexed. Enzymatic hydrolysis was carried out at 40 °C during 16 h. Finally, the volatile compounds released were determined using the SPME method detailed above.

#### 2.4.2. SPME-GC-MS Conditions

Separation, identification and semi-quantification of volatile compounds were performed on a GC 7820 A gas chromatograph (Agilent Technologies, Santa Clara, CA, USA) coupled with a 5975 Series MSD, Agilent mass spectrometer detector. The GC-MS system was equipped with an ZB-Wax column (Phenomenex; 60 m × 0.25 mm × 0.25 µm film thickness). For the SPME injections, the temperature of the column began at 40 °C and was held for 5 min, increased 3 °C·min^−1^ up to 220 °C. The constant column flow was 1.2 mL·min^−1^, using hydrogen (99.995%) as a carrier gas, and the injection port was at 250 °C. Mass spectra were scanned at 70 eV over a mass range from *m*/*z* 10 to 1000.

#### 2.4.3. Identification and Semi-Quantification Compounds

All volatile compounds were identified by comparing their retention indices (RI), retention times (RT) and mass spectra (authentic available standards and Willey library and NIST Mass Spectral Search Program (ChemSW Inc., NIST 98 Version Database, Gaithersburg, MA, USA). Compounds were semi-quantified as internal standard equivalents, using 3-octanol for: terpenes, ketones, aromatic hydrocarbons, lactones, norisoprenoids and sesquiterpenes, and 4 metil-2-pentanol for C_6_ compounds, aldehydes, acids, esters, alcohols and thiols.

#### 2.4.4. Statistical Analysis: Chemometric Tools

Instrumental data were analyzed using XLstat-Basic+ (Addinsoft, Paris, France). One-way analysis of variance (ANOVA) was applied to establish whether a significant difference (*p* ≤ 0.05; *p* ≤ 0.01; *p* ≤ 0.001) existed between the values obtained for the mean value of each parameter in the samples analyzed. The multiple range test (Fisher’s least significant difference method) was applied to confirm the results obtained.

Principal component analysis (PCA) on family volatile profiles of free and glicosidically bound fractions was applied to attempt the degree of separation among varieties studied and trying to identify similarities and differences among them. To obtain family volatile profiles the percentages of the different families of compounds, based on three-year mean values, were evaluated.

## 3. Results and Discussion

### 3.1. Environmental Conditions

Table S1 shows the environmental conditions (2015–2017) in the experimental vineyard. The Huglin index (HI) did not vary against years showing a warm climate (HI + 2) with cold nights (CI + 1) in 2015 and very cold nights (CI + 2) in both 2016 and 2017 in accordance with the Géoviticulture Multicriteria Climatic Classification System (GCCCMS) [35].

The year 2016 showed more days with temperatures higher than 35 °C while 2015 and 2017 had the same number of these hot days, lower than those of 2016. It should also be noted that 2016 was the rainiest year, with almost double rainfall than in 2015 and a quarter more than in 2017.

These differences in the environmental conditions throughout the three years studied could help to explain the differences in the concentration of varietal volatile compounds of each grape.

### 3.2. Oenological Parameters

Table 1 shows the values of grape maturity parameters determined in the three vintages studied. Each variety was harvested at different dates according to its optimal technological maturation stage and healthy state. ANOVA found significant differences for each parameter studied except for the pH, with lower significant differences for malic, tartaric and titratable acidity than for total soluble solids (TSS, ºBrix) and sugar. Average sugar content ranges from 179.20 g·L^−1^ in ‘Loureira’ up to 251.30 g·L^−1^ in ‘Verdello Blanco’. Average titratable acidity ranged from 5 g·L^−1^ in ‘Castañal’ up to 10.07 g·L^−1^ in ‘Caíño Tinto’.

The must obtained from ‘Albariño’, ‘Caiño Longo 1’, ‘Verdello Blanco’, ‘Caiño Blanco’, ‘Sousón’ and ‘Castañal’ grapes showed the highest TSS values with statistical differences with the rest of the varieties. In turn, ‘Loureira’ differed due to its low significant TSS value. In terms of total acidity, ‘Caiño Tinto’, showed a significant high content whereas ‘Castañal’ was the grape variety with a significantly lower value. ‘Caiño Bravo’, ‘Caiño Longo 2’ and ‘Caiño Tinto’ stood out for their greater relationship tartaric:malic acid showing varieties suitable for producing quality red wines. ‘Caíño Tinto’, ‘Loureira’ and ‘Caiño Longo 2’ were the varieties with a lower industrial maturity index according to the De Cillis and Odifredi maturation index compared to ‘Brancellao Blanco’, ‘Caiño Blanco’, ‘Castañal’ and ‘Sousón’, which would show better levels of maturation, since the range of industrial maturity is between 3 and 5 points depending on the variety considered. In general, the harvest with the highest TSS was in 2015 followed by 2017, while 2017 showed the highest values of total acidity, probably due to the climatic events that took place in 2017. It can be seen in Table S1 that 2017 had the lowest values according to the cold night index and had the lowest minimum temperatures. In this vintage, vineyards in the area suffered critical frost in April, which generated a big loss of the shoots born to date. Nevertheless, it was followed by a strong regrowth and hence the harvest continued under very favorable weather conditions.

### 3.3. Volatile Composition

A total of 339 volatile compounds were identified among the three vintages studied, 230 compounds were identified as free forms and 205 as aromatic precursors. They were grouped in thirteen volatile families: acids, alcohols, C_6_ compounds, aldehydes, esters, phenols, polycyclic aromatic hydrocarbons (PAH’s), lactones, C_13_-norisoprenoids, sesquiterpenes, terpenes and thiols.

Table 2 and Table 3 show that C_6_ compounds were the family with the highest contribution in all grape varieties in free form, whereas alcohols, phenols and terpenes did it at an aromatic precursor level (Table 4 and Table 5).

In terms of the number of compounds, white varieties showed a higher number of acids, PAH’s, C_13_-norisoprenoids and terpenes, while red varieties showed a higher number of alcohols, C_6_ compounds, aldehydes, esters and lactones in both free and bound fractions.

#### 3.3.1. Free Volatile Compounds

Table 2 and Table 3 show, for white and red varieties, respectively, the volatile composition (2015–2017) for each volatile family in free form. To complete the discussion of the results obtained, Table 6 and Table 7 list which compound was the most abundant in each grape variety and if there were common compounds among them.

Results did not show significant differences according to the grape variety. ‘Caiño Blanco’ showed a total content of 2980 µg·L^−1^ compared to ‘Brancellao’ that reached up to 9460 µg·L^−1^. In a previous study, Vilanova et al. [36] found that the monovarietal wine from ‘Caiño Longo’ showed the highest content of free compounds, followed by ‘Caiño Tinto’ and ‘Caiño Bravo’. Similar results were obtained in this study.

In order to determine the higher or lower aromatic potential of the twelve grape varieties, a more detailed description was carried out for each volatile family.

The contribution (%) of each family of volatile compounds in free form to the aromatic profile of every grape variety is shown in Tables S2 and S3.

C_13_-norisoprenoids were detected in free form in all grape varieties studied except ‘Brancellao’. This group of compounds had a higher content in the white varieties, ‘Caiño Blanco’ being the variety that showed the highest value. C_13_ -norisoprenoids are varietal aroma compounds positive to the aroma of wines [37] because of their powerful odor, as the result of their low odor threshold [38]. In this study, α-damascenone was the major norisoprenoid in most of the studied varieties (Table 6 and Table 7). β-damascenone was also present in ‘Caiño Blanco’, ‘Caiño Bravo’, ‘Sousón’ and ‘Verdello Blanco’. ‘Loureira’ was the grape variety that showed the highest number of different C_13_-norisoprenoids identified, with α-ionone, α-damascenone, δ-damascone, ionone and β-damascone.

The importance of this group of volatile compounds is also due to their positive aroma descriptor, such as sweet, floral, and fruity for α-damascenone [39], and sweet and apple nuances [40] for β-damascenone. β-ionone is defined with different descriptors such as floral, dark berry or red berry aroma [37] and violet aroma [41], depending on the wine matrix. The presence of α-ionone together with β-ionone is associated with sweet floral notes [42].

Terpenes are important grape-derived compounds mainly responsible for the characteristic aroma of muscat or related aroma wines, but also of other non-muscat varieties [28]. Relative recent published data showed the wide range of monoterpene content among different varieties and wines, a reason why there are thought to be good varietal compounds [43]. The presence and content of terpenes were also applied to differentiate grapevine varieties using them as varietal markers. In this study, ‘Loureira’, ‘Verdello Blanco’ and ‘Albariño’ were the white grape varieties with the higher terpene content and also showed a higher number of different terpenoid forms, 46, 43 and 31, respectively. Nevertheless, it should be considered that probably only a part of those terpenes will have an important implication in wine aroma [44].

Among the red varieties, ‘Caiño Longo 2’ showed the highest terpene level as well as a high terpene compound diversity, with 27 different compounds identified along the three years studied. Total average terpene concentrations per variety are shown in Table 2 and Table 3.

Table 6 and Table 7 show that α -terpineol, with a descriptor of anise [41], was the terpene that reached the highest concentration in ‘Albariño’, ‘Caiño Blanco’, ‘Caiño Longo 2’ and in ‘Caiño Bravo’. In this last variety, α -terpineol was present in similar content to nerol, that was indeed the major one. Similar results for this variety were obtained by Vilanova et al. [36] and Oliveira et al. [45] of this terpene in free and glycosidic fraction, respectively. Terpenyl acetate was the main terpenoid compound identified in ‘Caiño Tinto’, ‘Castañal’ and ‘Sousón’ while α -citronellol, with a citrus descriptor, was identified as the highest concentration terpene in ‘Caiño Longo 1’. Linalyl anthranilate was the terpene with the highest content in ‘Brancellao’ and ‘Loureira’. This compound is described with fresh linalool, gardenia and orange-like aroma [46]. Nerol was the terpene with the highest concentration in ‘Brancellao Blanco’, 35 µg·L^−1^, with rose, fruity and floral descriptors [44]. Finally, ‘Verdello Blanco’ showed linalool as the highest concentration terpene, characterized with floral, lavender and citrus odor [44]. Most of these terpenes were already highlighted by Marais, J. [28] for being the most prominent terpenes in aroma-related cultivars.

Sesquiterpenes (C_15_) are the other major important terpene group with balsamic, spicy and woody aroma descriptors [47]. Nevertheless, their contribution to the wine and grape aroma is low, given that their concentrations are usually below their corresponding odor threshold [48]. Sesquiterpenes have also been added in functional foods or cosmetics due to their bioactive and preservative health properties [49]. In this study, it was only α-calacorene in ‘Caíño Tinto’ that was detected in the free fraction. Nevertheless, it showed a high interannual variability as it was only detected in 2017. This terpene was also previously identified in ‘Baga’ ripe grapes by Coelho et al. [50] and in the skin of ‘Bual’ and ‘Bastardo’ grapes by Perestrelo et al. [51], reported for exhibiting a woody aroma.

Organic acids, together with terpenoids, precursors of aromatic esters, aldehydes and thiols are some of the most important families of volatile compounds. Organic acids could provide sourness to the wines or even rancid or pungent aromas [52]. In this study, ‘Verdello Blanco’ stood out for its high concentration, facing ‘Brancellao Blanco’ with the lowest value. However, it should be noted the high standard deviation shown in ‘Verdello Blanco’ could point out an outlier value that stands out over the other two vintages. Hexanoic acid, with rancid, grass and fruity descriptors, was the major acid identified in almost every variety studied, such as ‘Verdello Blanco’ among the white varieties (Table 6) and ‘Sousón’ among the red ones (Table 7), coinciding these last results with those previously obtained by Canosa et al. [53]. Octanoic and nonanoic acid were two other acids identified in all grape varieties studied.

C_6_ compounds and alcohols were the volatile compounds with the higher total concentration in free form. Alcohols showed their lowest values in ‘Caíño Tinto’ with a total content of 651.62 µg·L^−1^, with ‘Verdello Blanco’ once again being the variety with the highest mean concentration, 1615 µg·L^−1^, highlighting its high standard deviation. Higher alcohols, being one of the most quantitatively important family compounds in grapes, allow us to explain some aromatic part of the future wines, such as structure and vinous characteristics [54]. They generally have solvent odor descriptors with some exceptions, such as 2-phenylethanol which is characterized by a floral, rose-like odor. Its level in wine is associated with the type of grape variety used, as it is one of the main derivatives of the phenylpropanoid metabolism [55]. In this study, 1-hexanol, 2-ethyl was the most abundant volatile in all varieties, except for ‘Castañal’ with *cis*-2-ethyl-2-hexen-1-ol (Table 6 and Table 7). These compounds could be responsible for herbaceous or green aromatic notes. Regarding C_6_ compounds, the lowest values were observed in ‘Caíño Blanco’ with 1305 µg·L^−1^ compared to ‘Brancellao’ which showed the highest value of 5876 µg·L^−1^. C_6_ compounds include alcohols and aldehydes that are derived from the lipidic membrane of grapes. As those compounds are derived from varietal precursors, they have been tested as varietal markers with monovarietal wines with some preliminary successful results [24]. They generally receive green and grass descriptors, including those in the herbaceous odor series [56]. ‘Brancellao’ showed the highest C_6_ compound concentration with 2-hexenal, (*E*) as its major compound, being possibly the most herbaceous-like variety in this study. Alternatively, ‘Castañal’ and ‘Sousón’ showed 1-hexanol as their main C_6_ compound, with the ‘Sousón’ results coinciding with those obtained by Canosa et al. [53] (Table 7). Among the white varieties, high values were also shown in ‘Albariño’ by López-Tamames et al. [57], which specially relates C_6_ compounds such as hexanal, (*E*)-hex-3-en-1-ol and (*E*)-hex-2-en-1-al with herbaceous notes, usually related with the maturation index of the grapes. Generally, grape varieties with higher acidity levels show a higher concentration of these volatile compounds [57].

Aldehydes were found among a narrow range of values in the free fraction in almost every grape variety studied, with the exception of ‘Brancellao’ with an outlier value of 629.34 µg·L^−1^. However, the high standard deviation shown implies a high interannual variability. C_6_ and C_9_ aldehydes were originated from the oxidation process of fatty acids contained in grapes, as linoleic and linolenic acid, which could be responsible for herbaceous aromas [58]. In the free fraction, benzaldehyde, 2,5-dimethyl-, benzaldehyde and (*E*)-2-nonenal were the major aldehydes identified (Table 6 and Table 7), some of which have been reported to be compounds of significance in Noble muscadine wine. Benzaldehyde, 2-phenylethanol and methyl salicylate are primarily derived from phenylalanine [29]. Acetaldehyde, phenyl-, stood out as the major aldehyde in ‘Caiño Tinto’, being associated with hawthorn, honey and sweet descriptors [59].

Esters and phenols were detected in low concentrations in the grapes studied. Only two varieties, ’Caíño Blanco’ and ‘Loureira’, showed higher concentrations of esters, while ‘Castañal’ and ‘Brancellao’ showed higher concentrations of phenols. Nevertheless, standard deviations could be seen, possibly because of the interannual differences. Esters are related to the fruity character of wines [38] and those with black and red berry fruity notes are usually very important to red wine global aroma [27]. It is known that one of the differential factors in wine ester quantities will be the must composition of the grape variety [27]. Indeed, Cabaroglu et al. [60] also referred to specific grape cultivar esters. Pérez-Navarro et al. [58] considered that the wine ester diversity is a consequence of the genotypic varietal diversity of grapes used, explained by the availability of lipids in each variety.

Regarding phenols, estragole was identified in all grape varieties with concentrations that ranged from 2 µg·L^−1^ in ‘Loureira’ and ‘Sousón’ up to 27µg·L^−1^ in ‘Verdello Blanco’. This compound is associated with anise-like and herbaceous descriptors [61,62]. Caven-Quantrill and Buglass [62] referenced the presence of estragole, together with (*E*)-anethole and methyl salicylate in the must of the ‘Madeleine Angevine 7672’ grape variety. 2,6 di-tert-butyl-p cresol (BHT) was the other phenol identified in almost all studied varieties but only in 2017. This compound was also previously identified in sherry wines [63]. *P*-cresol is a volatile compound present in smoke and it was also identified in some wines vinified with grapes exposed to smoke that could even give them an ashy taste [64]. Considering the fact the area where the experimental vineyard is located suffered an important wave of fires in the 2017 vintage, it could be viable t think that the smoke has impregnated the grapes remaining up to their harvest. In the white grape varieties (Table 6), 2,4-di-tert-butyl- and phenol,4-ethyl- were the major phenols, 2,6-bis(1,1-dimethylethyl)-4-methyl- being the major phenol in the red ones, except for ‘Caiño Bravo’ and ‘Caiño Longo 1’ where estragole was the most abundant (Table 7).

All varieties showed similar concentrations for ketones and PAH’s, except ‘Brancellao Blanco’, which stood out for the lowest ketone values and ‘Castañal’ for the highest PAH content. Carbonyl compounds, such as ketones, are associated with rancidity and herbaceous odors and their origin is associated with lipoxygenase activity of the grape and must aeration [65]. Sulcatone has been identified in all studied varieties, its contribution mainly being important in the white grapes (Table 6). This compound was also identified by Furdíková et al. [66] in Gewürztraminer wines and described as fruity, apple-like or creamy. With respect to the red grapes, acetophenone was the major ketone identified (Table 7).

Lactones were detected in free form in all varieties. This group of volatile compounds is characterized by a fruity aroma, despite the fact that their sensorial characteristics are influenced by their chemical structure [67]. Among them, butyrolactone, one of the better-known lactones was detected in ‘Brancellao’, ‘Loureira’ and ‘Caiño Longo 2’. It has creamy and fatty descriptors [66] and can be found in mg·L^−1^ in wines despite the fact that it is not likely to produce any major effects on their organoleptic characteristics [25]. Tetrahydrofuran-2-one,4,4,5,5-tetramethyl was identified in the majority of the grape varieties studied except for ‘Loureira’ and ‘Verdello Blanco’. ‘Caiño Blanco’ and ‘Loureira’ were those white varieties showing the highest concentrations.

Finally, with respect to thiols, they were only detected in free form in 4 out of the 12 varieties studied. ‘Albariño’ and ‘Brancellao Blanco’ among the white varieties and ‘Castañal’ and ‘Sousón’ among the red ones. This volatile family also showed a high interannual variability. Thiols, such as 3-mercaptohexyl acetate (3MHA, 2) and 3-mercaptohexan-1-ol (3MH, 3), are considered varietal aromas because and they are the result of the odorless precursor cleavage compounds presented in grapes [68]. Volatile thiols could be derived from synthesis processes in which C_6_ aldehydes could participate [69]. In wine, thiols were initially associated with negative odors, but later started to also be related to different herbaceous, mineral and fruity aromas of wine [70].

#### 3.3.2. Aromatic Precursor Fraction

Table 4 and Table 5 show, for the white and red varieties, respectively, the volatile composition (2015–2017) for each volatile family in bound form. Table 6 and Table 7 list which compound is the most abundant in each grape variety and also if there are common compounds among them.

The contribution (%) of each family of volatile compounds in bound form to the aromatic profile of every grape variety is shown in Tables S4 and S5.

Despite the fact that numerous grape berries, specially *Vitis vinifera* L., have no odor, their wines still present a characteristic varietal aroma [71]. This is due to the aroma glycosides contained in grapes, above all in those non-aromatic varieties, in which glycosidic aromatic compounds are an important source of potential aroma volatiles that would contribute to the wine aroma profile [72].

A broad range of concentration values were also identified among the different aromatic precursor families. ‘Brancellao’ was the variety with the lowest total concentration, 16,199 µg·L^−1^ compared to ‘Caiño Longo 2’, with 80,736 µg·L^−1^, which showed the highest total concentration value. The most abundant volatile families in bound fraction were alcohols, phenols, terpenes and esters.

C_13_-norisoprenoids were more abundant in the free fraction than under bound form. Among the white varieties, ‘Albariño’ showed the highest total concentration of C_13_-norisoprenoids followed by ‘Loureira’ and ‘Caiño Blanco’ (Table 4). Whereas in the red varieties, C_13_-norisoprenoids in ‘Caiño Longo 2’ were only identified (Table 5). This grape variety also showed the highest concentration of C_13_-norisoprenoids in free form. α-damascenone was identified in ‘Albariño’ and ‘Caiño Longo 2’ while dihydro-β-ionol was detected in ‘Loureira’ and ‘Caiño Blanco’, with a woody, flowery and camphoraceous odor [46].

Results in Table 4 and Table 5 also showed that there was an important increase in the concentration and number of terpenes in all varieties with respect to their corresponding free fraction. This is very interesting to show the notable importance of the hidden compounds in the aromatic potential of certain grapes. Results obtained in previous research by Black et al. [44] showed that terpene glycoside concentration exceeds the free terpenes one being the bound: free terpene ratio higher than 1 in all grapevine varieties studied [73]. Unfortunately, this glucoside aromatic potential usually keeps rather stable during the winemaking process [74]. In general, white varieties show higher terpene levels than the red ones [26]. Similar to the free fraction, in bound form, ‘Loureira’, ‘Verdello Blanco’, ‘Albariño’ and ‘Caiño Longo 2’ were those varieties with a higher number and total terpene concentration, linalool being the major terpene in all of them (Table 6 and Table 7). Diéguez et al. [34] also identified linalool as the major terpene in ‘Albariño’ in its bound form and Oliveira et al. [45] in ‘Loureira’ and as the second major one in ‘Albariño’. In ‘Verdello Blanco’ and ‘Loureira’, the second highest terpene was tetrahydro linalool with sweet, oily and floral odor [46]. In ‘Albariño’, the presence of 3-octanol, 3,6-dimethyl- was very important, also known as AR1. In ‘Caiño Longo 2’, the presence of α-terpineol is notable, with anise notes, and was also the major terpene in the free fraction of this variety. Thymol was the major terpene in ‘Brancellao’, ‘Caiño Bravo’, ‘Caiño Longo 1’ and ‘Sousón’ (Table 7).

Three sesquiterpenes were detected in the bound form. Patchoulane, a tricyclic structure sesquiterpene, was detected in ‘Albariño’, ‘Brancellao’, ‘Brancellao Blanco’, ‘Caiño Blanco’, ‘Caiño Longo 2’ and ‘Verdello Blanco’. This compound was also identified in Merlot wines by Welke et al. [75]. Ledene oxide-(II) and isopatchoulane were also identified in ‘Caiño Longo 2’.

Regarding the organic acids, ‘Albariño’ was the grape variety with higher contribution of this volatile family in bound form. It showed an average concentration value of 3040 µg·L^−1^ compared to the red variety ‘Caíño Bravo’ with 15.08 µg·L^−1^, which additionally showed a high standard deviation. Acids in bound form showed higher contents in white grapes, except for ‘Verdello Blanco’ (Table 4), while different behaviors were appreciated among the red grape varieties studied (Table 5). ‘Brancellao’, ‘Caiño Bravo’, ‘Caiño Tinto’ and ‘Sousón’ showed lower content than in the free fraction, whereas ‘Caiño Longo 1’, ‘Caiño Longo 2’ and ‘Castañal’ increased their corresponding values. Octanoic and nonanoic acid were the two major acids found, both also being abundant in the free fraction (Table 6 and Table 7). As it was previously mentioned, the octanoic acid has fatty acid, dry and dairy descriptors [76].

C_6_ compounds generally showed higher concentrations in the bound fraction of red varieties, highlighting ‘Sousón’ for its quite high concentration of 6381 µg·L^−1^. It was identified as the lowest concentration in ‘Caíño Blanco’ with 1156 µg·L^−1^. 1-hexanol was the compound that showed the highest concentration in all varieties in the bound fraction (Table 6 and Table 7).

All red and white varieties showed a higher concentration of bound alcohol compounds, ‘Loureira’ being the one showing the highest concentration with 21,216 µg·L^−1^, compared to ‘Brancellao’ with an average concentration of 8127 µg·L^−1^. A high concentration of alcohols is desirable because they have a great trend of producing fruity esters with the presence of carboxylic acids along the vinification process [52]. It should be highlighted that the major alcohols identified were: 2-phenyl ethanol and benzyl alcohol. Both benzyl and phenyl derivatives were found to be the major compounds in the bound fraction of numerous neutral grape varieties [2,26]. 2-phenyl ethanol is characterized by rose [77,78,79,80,81], honey [78,80] and floral [82] descriptors and benzyl alcohol is characterized by roasted, toasted [78,80], sweet and fruity descriptors [82]. Among the white varieties, benzyl alcohol was the major alcohol in ‘Brancellao Blanco’ and in ‘Albariño’, with a similar content of 2-phenyl ethanol in accordance with the results obtained by Diéguez et al. [34] in 1997 (Table 6). Benzyl alcohol was also the major alcohol in ‘Brancellao’, ‘Caiño Bravo’ and ‘Caiño Tinto’ among the red ones (Table 7). Vilanova et al. [36] highlighted its concentration in ‘Caiño Bravo’ wines as it could provide blackberry notes to this variety [83]. 2-phenyl ethanol was the major alcohol in ‘Caiño Blanco’, ‘Loureira’ and ‘Verdello Blanco’ among the white ones (Table 6) and in ‘Caiño Longo 1’, ‘Caiño Longo 2’, ‘Castañal’ and ‘Sousón’ among the red ones (Table 7).

Only four aldehydes were detected in bound form, lower than in free form but in significantly higher content mainly in red grape varieties (Table 5). Benzaldehyde was the major aldehyde detected, with an almond-like and bitter volatile compound descriptors [84] (Table 6 and Table 7).

Compared to what happened in the free fraction with esters and phenols, in the bound fraction, a higher number of different esters were identified and in a higher concentration. It should be noted that ‘Albariño’ and ‘Caiño Longo 2’ are those varieties with the highest values, with nonanoic acid ethyl ester and nonanoic acid methyl ester, respectively, compared to ‘Castañal’ showing the lowest concentration. Methyl salicylate, with mint and green-like flavored odors and its wintergreen characteristic aroma [85], was the major ester in ‘Brancellao’, ‘Caiño Blanco’, ‘Caiño Bravo’, ‘Caiño Longo 1’, ‘Caiño Tinto’, ‘Sousón’ and ‘Verdello Blanco’ (Table 6 and Table 7). This compound, together with acetaldehyde, benzyl alcohol or (*E*)-2-octenal, was reported as important in Noble muscadine wine flavor [29]. It also seems to be a biomarker of downy mildew infection in grapevines, and even a negative relationship between the emission of some volatile organic compounds (VOCs), such as methyl salicylate, and the severity of the disease could suggest a new role of those VOCs against plant diseases [86]. Nevertheless, it was also identified in healthy grapes by Poitou et al. [85].

Phenols’ family showed, in general, lower concentrations in the red varieties except for ‘Caiño Longo 2’, which was the variety with the highest content. While in free form, only two phenols were identified and up to 10 different compounds were identified in the bound fraction. 2,4-di-tert-butylphenol was the major one in almost every variety, with the exception of ‘Brancellao’ with 4-propylguaiacol or ‘Brancellao Blanco’ and ‘Verdello Blanco’ with phenol, 4-ethyl- (Table 6 and Table 7).

Referring to ketones, there was a general increase in the bound fraction while there was a reduction in the PAH concentration regarding the free one. Methanone, diphenyl- was the major ketone in ‘Albariño’, ‘Caiño Longo 2’ and ‘Castañal’, also being detected the acetophenone in these varieties. ‘Verdello Blanco’ did not have acetophenone and its highest ketone was 3-otanone in ‘Brancellao Blanco’ (Table 6).

Regarding lactones, only γ-undecanolactone was detected in ‘Caiño Longo 2’ in the bound fraction, the variety which also showed the highest concentration of lactones in the free form. This lactone has already been identified in sparkling wines from Croatia by Korenika et al. [87] with fruity, peach and creamy notes [39].

The same two thiol compounds, nonanal, 3-(methylthio)- and 2-undecanethiol,2-methyl-, were identified in the bound fraction and in higher concentrations, except in ‘Verdello Blanco’.

#### 3.3.3. Volatile Relationship between Varieties

With the main objective of better visualizing the results obtained in the free and bound volatile compound determination and checking if it is possible to find any relationship among varieties based on their aromatic profile, two principal components analyses (PCA) were performed.

The ACPs were made with the percentages of each family, according to studies on other types of compounds, such as phenolics, in which the influence of non-genetic factors, such as environment factors, on their content was more important than on their qualitative profile [88,89,90].

Taking into account the previous hypothesis, the present study aims to verify the same behavior with aromatic compounds and thus reduce the influence of external factors on the varietal aromatic profile, seeking to limit the results as much as possible to those triggered by their genetic influence.

**Figure 1:**
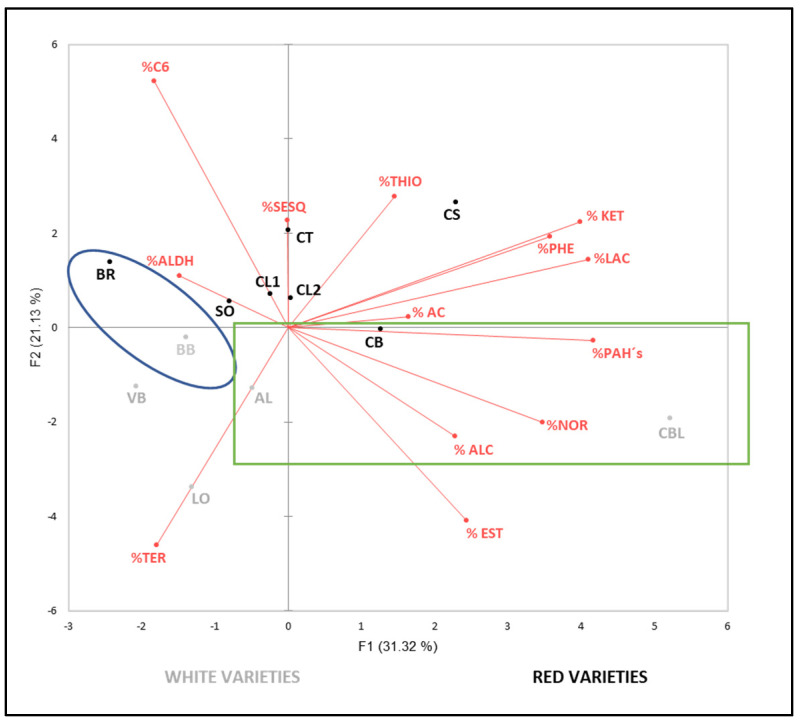
Principal component analysis on free aromatic fraction profile (percentage) of grapes. NOR: norisoprenoids, AC: acids, EST: esters, KET: ketones, PAHs: polycyclic aromatic hydrocarbons, ALC: alcohols, ALDH: aldehydes, C6: compounds C6, THIO: thiols, SESQ: sesquiterpenes, PHE: phenols, TER: terpenes, LAC: lactones. AL: ‘Albariño’, BR: ‘Brancellao’, BB: ‘Brancellao Blanco’, CBL: ‘Caíño Blanco’, CB: ‘Caiño Bravo’, CL1: ‘Caiño Longo 1’, CL2: ‘Caiño Longo 2’, CT: ‘Caiño Tinto’, CS: ‘Castañal’, LO: ‘Loureira’, SO: ‘Sousón’ and VB: ‘Verdello Blanco’.

**Figure 2:**
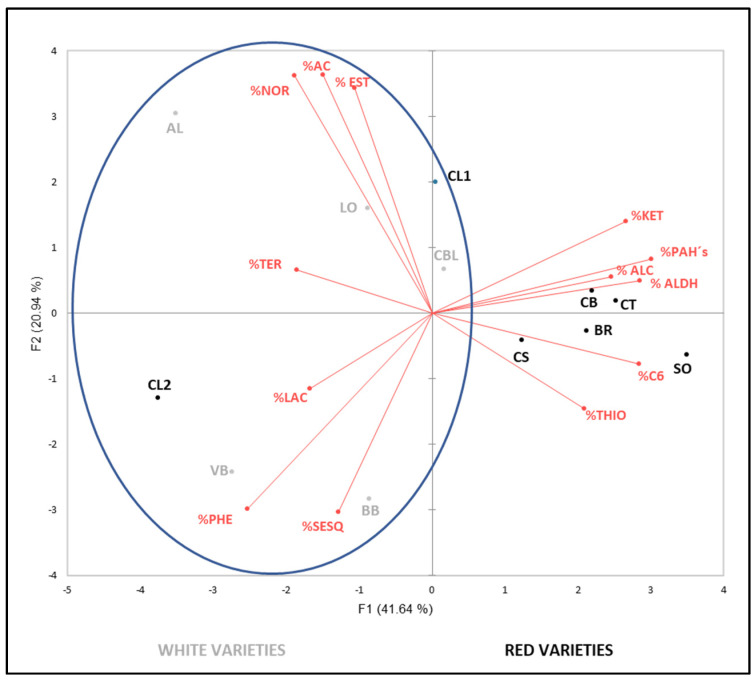
Principal component analysis on aromatic precursor faction profile (percentage) of grapes. NOR: norisoprenoids, AC: acids, EST: esters, KET: ketones, PAHs: polycyclic aromatic hydrocarbons, ALC: alcohols, ALDH: aldehydes, C6: compounds C6, THIO: thiols, SESQ: sesquiterpenes, PHE: phenols, TER: terpenes, LAC: lactones. AL: ‘Albariño’, BR: ‘Brancellao’, BB: ‘Brancellao Blanco’, CBL: ‘Caíño Blanco’, CB: ‘Caiño Bravo’, CL1: ‘Caiño Longo 1’, CL2: ‘Caiño Longo 2’, CT: ‘Caiño Tinto’, CS: ‘Castañal’, LO: ‘Loureira’, SO: ‘Sousón’ and VB: ‘Verdello Blanco’.

Figure 1 and Figure 2 show both ACP plots corresponding to the volatile profiles of the 12 varieties, in free and bound form, respectively. They were carried out with the average percentage of the three vintages studied (2015–2017) for each volatile family.

The first PCA, Figure 1, was carried out with the percentage values of the free fraction volatile families. The first two principal components explained 52.45% of the total variance (31.32% and 21.13%, respectively). F1 was primarily characterized by the contribution of PAHs, ketones, C_13_-norisoprenoids and phenols. F2 was characterized by C_6_ compounds and terpenes. White varieties, together with ‘Caiño Bravo’, were situated in the negative part of F2, principally by their higher terpene proportion in ‘Albariño’, ‘Loureira’, ‘Brancellao Blanco’ and ‘Verdello Blanco’ and the lower C_6_ compound proportion in ‘Caiño Bravo’ and ‘Caiño Blanco’. At the same time, it should be noted that ‘Caíño Blanco’, considered to be the result of a cross among ‘Caiño Bravo’ and ‘Albariño’ [17], shows a higher aromatic similarity with ‘Caiño Bravo’ than with ‘Albariño’, with a considerably lower terpene proportion than ‘Albariño’, showing more similar percentages of acids, alcohols, ketones, PAHs and lactones with ‘Caiño Bravo’ (Tables S2 and S3). These results could cause a higher genetic implication of ‘Caiño Bravo’. This variety is equally different from the rest of the red varieties by F2 because of its lower percentage of C_6_ compounds, with a proportion lower than 50% facing 60% in most of the red varieties. Despite ‘Caiño Bravo’ showing one of the lowest ºBrix degrees, which apparently could be different to this result, it also showed the second lowest tartaric acid rate. Another interesting result in this PCA is the proximity between ‘Brancellao’ and ‘Brancellao Blanco’, which could support the already outlined theory by [1] that ‘Brancellao Blanco’ is a persistent color mutation of ‘Brancellao’.

The second PCA, Figure 2, was carried out with the percentage values of the aromatic precursor volatile families. The first two principal components explained 62.58% of the total variance (41.64% and 20.94%, respectively). F1 was primarily characterized by the PAHs, ketones and aldehydes. F2 was characterized by esters, phenols, norisoprenoids and acids. As it happened in the previous PCA, a good differentiation among varieties could be appreciated. Once again, F1 separates white and red varieties, with a few exceptions: ‘Caiño Blanco’ was separated again from the rest of white varieties. In the same way, ‘Caiño Blanco’ appeared nearer to ‘Caiño Bravo’ than to ‘Albariño’, which could be seen in more detail in the aromatic precursor percentages (Tables S4 and S5) This could result from the possible theory that ‘Caiño Bravo’ has a higher genetic influence than ‘Albariño’ in ‘Caiño Blanco’. Red variety of ‘Caiño Longo 2’ moves away from the rest of the red varieties, mainly because of its high phenol and terpene proportions, which generally occurs in white varieties.

## 4. Conclusions

A detailed and broad study of the aromatic profile of 12 minority varieties was carried out. The high number of differences among the different varieties’ aromatic profiles reinforces the importance of characterizing them from an aromatic point of view.

Among the red varieties studied, ‘Caiño Bravo’ could be highlighted for its potential glycosidic terpene concentration, which because of a high acidity rate and a longer vegetative cycle could be interesting in terms of facing the effects of climate change. Among the white varieties, ‘Caiño Blanco’ was also different because of its higher norisoprenoid concentration, as well as longer vegetative cycle, achieving a good sugar–acidity ratio over time. This could make it a good variety to provide freshness and aromaticity in blends with other varieties with shorter cycles and less acidity.

Varieties such as ‘Verdello Blanco’ and ‘Caiño Longo 2’ were newly characterized and from the results obtained, they seem to be aromatic varieties. Nevertheless, several differences were shown between years, which is the reason why it is thought that these results should be confirmed with more years of study.

The results obtained showed that the determination of varietal compounds is an important tool to differentiate and classify varieties, in addition to making an approximation between varieties that are believed to be genetically related. For example, a permanent mutation, as in the case of ‘Brancellao’ and ‘Brancellao Blanco’ or a paternity relationship, as in the case of ‘Caiño Bravo’ and ‘Albariño’ with ‘Caiño Blanco’.

From an applied point of view, the data obtained could allow wine growers and oenologists to choose a higher varietal diversity, also providing information to develop the most adequate management depending on the type of variety and their elaboration objectives, which could increase the range of differentiation in this winemaking area.

In order to consider the varietal aroma profile as a good chemo-taxonomic tool, it is necessary to complete the results obtained in this research with a deeper analysis of the individual compounds, including more grape varieties from different genetic groups, which allows the possibility of developing a broader data network.

## Figures and Tables

**Table 1:** Physicochemical parameters of the grapes in 2015, 2016 and 2017 vintages.

Variety	Ab.	Collecting Data	ºBrix	Sugar (g·L^−1^)	Total Acidity (g·L^−1^)	pH	Malic Acid (g·L^−1^)	Tartaric Acid (g·L^−1^)	MI:CO	MI:BS	T:M
‘Albariño’	AL	27/08/2015	23.7	234.3	8.6	3.20	8.3	9.1	2.76	1.06	0.91
		14/09/2016	22.1	226.4	5.8	3.45	2.6	6.3	3.81	1.09	0.41
		13/09/2017	22	214.7	7.8	3.22	4.5	5.5	2.82	0.71	0.82
			22.6 ± 0.95 d	225.1 ± 9.86 e	7.4 ± 1.44 abc	3.29 ± 0.14 a	5.1 ± 2.90 abc	7.0 ± 1.89 cd	3.13 ± 0.59 abc	0.95 ± 0.21 bc	0.71 ± 0.27 abc
‘Brancellao’	BR	16/09/2016	21.8	212.4	5.8	3.42	2.2	7.1	3.76	1.22	0.31
		13/09/2017	19.1	181.7	8.9	3.13	4.8	6.8	2.15	0.76	0.71
			20.5 ± 1.91 abcd	197.1 ± 21.71 abcd	7.4 ± 2.19 abc	3.28 ± 0.21 a	3.5 ± 1.84 ab	7.0 ± 0.21 cd	2.95 ± 1.14 abc	0.99 ± 0.33 bc	0.51 ± 0.28 abc
‘Brancellao Blanco’	BB	18/09/2015	19.2	182.9	4.6	3.90	2.3	5.6	4.17	1.22	0.41
		15/09/2016	19.3	184.0	5.5	3.35	1.1	7.0	3.51	1.27	0.16
			19.3 ± 0.07 ab	183.5 ± 0.78 ab	5.1 ± 0.64 ab	3.63 ± 0.39 a	1.7 ± 0.8 a	6.3 ± 0.99 bcd	3.84 ± 0.47 cd	1.25 ± 0.04 c	0.28 ± 0.18 a
‘Caíño Blanco’	CBL	08/09/2015	23.6	233.2	6.4	3.65	5.5	5.8	3.69	0.91	0.95
		19/09/2016	22.2	217.0	5.8	3.40	2.2	6.3	3.83	1.09	0.35
		11/09/2017	21.8	212.4	8.9	3.08	5.2	5.9	2.45	0.66	0.88
			22.5 ± 0.94 d	220.9 ± 10.93 de	7.0 ± 1.64 abc	3.38 ± 0.2 a	4.3 ± 1.82 ab	6.0 ± 0.26 abcd	3.32 ± 0.76 bcd	0.89 ± 0.21 abc	0.73 ± 0.33 abc
‘Caíño Bravo’	CB	21/09/2015	20.1	193.0	6.2	4.00	5.0	5.0	3.24	0.81	1.00
		28/09/2016	18.6	176.1	7.2	3.46	5.4	4.1	2.58	0.57	1.32
		26/09/2017	21.0	203.3	8.7	3.26	6.6	4.1	2.41	0.47	1.61
			19.9 ± 1.21 abc	190.8 ± 13.73 abc	7.4 ± 1.26 abc	3.57 ± 0.38 a	5.7 ± 0.83 bc	4.4 ± 0.52 ab	2.75 ± 0.44 abc	0.62 ± 0.17 ab	1.31 ± 0.30 de
‘Caíño Longo 1’	CL1	18/09/2015	23.4	230.8	7.1	3.40	4.2	6.8	3.30	0.96	0.62
		15/09/2016	20.1	193.0	7.9	3.30	5.1	6.2	2.54	0.78	0.82
		13/09/2017	22.6	221.6	9.8	3.20	7.4	5.2	2.31	0.53	1.42
			22.0 ± 1.72 cd	215.1 ± 19.71 cde	8.3 ± 1.39 bc	3.30 ± 0.10 a	5.6 ± 1.65 bc	6.1 ± 0.81 abcd	2.72 ± 0.52 abc	0.76 ± 0.21 ab	0.95 ± 0.42 bcd
‘Caíño Longo 2’	CL2	21/09/2015	21.0	203.3	6.9	3.50	5.4	5.3	3.04	0.77	1.02
		15/09/2016	20.2	194.2	8.3	3.25	4.8	6.1	2.43	0.73	0.79
		27/09/2017	20.2	194.2	10.5	3.05	7.1	5.3	1.92	0.50	1.34
			20.5 ± 0.46 abcd	197.2 ± 5.25 abcd	8.6 ± 1.81 bc	3.27 ± 0.23 a	5.8 ± 1.19 bc	5.6 ± 0.46 abcd	2.47 ± 0.56 abc	0.67 ± 0.14 ab	1.05 ± 0.28 cde
‘Castañal’	CS	21/09/2015	22.4	219.3	4.6	3.80	3.0	5.3	4.87	1.15	0.57
		28/09/2016	21.3	206.7	4.4	3.62	1.9	3.8	4.84	0.86	0.50
		26/09/2017	21.8	212.4	6.0	3.44	3.3	4.0	3.63	0.67	0.83
			21.8 ± 0.55 cd	212.8 ± 6.31 cde	5.0 ± 0.87 a	3.62 ± 0.18 a	2.7 ± 0.74 ab	4.4 ± 0.81 a	4.45 ± 0.71 d	0.89 ± 0.24 abc	0.63 ± 0.17 abc
‘Caíño Tinto’	CT	22/09/2015	19.2	182.9	8.7	3.40	7.4	5.1	2.21	0.59	1.45
		27/09/2016	18.7	177.2	7.6	3.40	5.3	4.8	2.46	0.63	1.10
		27/09/2017	19.5	186.2	13.9	3.03	10.8	5.1	1.40	0.37	2.12
			19.1 ± 0.40 ab	182.1 ± 4.55 a	10.1 ± 3.37 c	3.28 ± 0.21 a	7.8 ± 2.78 c	5.0 ± 0.17 ab	2.02 ± 0.55 a	0.53 ± 0.14 a	1.56 ± 0.52 e
‘Loureira’	LO	21/09/2015	17.4	162.7	8.0	3.20	3.1	8.6	2.18	1.08	0.36
		20/09/2016	18.4	184.1	7.5	3.07	2.0	7.5	2.45	1.00	0.27
		26/09/2017	19.9	190.8	7.9	3.18	4.5	5.9	2.52	0.75	0.76
			18.6 ± 1.26 a	179.2 ± 14.68 a	7.8 ± 0.26 abc	3.15 ± 0.07 a	3.2 ± 1.25 ab	7.3 ± 1.36 d	2.38 ± 0.18 ab	0.94 ± 0.17 bc	0.46 ± 0.26 ab
Sousón’	SO	28/09/2016	20.5	197.6	6.8	3.37	3.6	4.9	3.01	0.72	0.73
		26/09/2017	22.6	221.6	5.6	3.61	3.5	4.3	4.04	0.77	0.81
			21.6 ± 1.49 bcd	209.6 ± 16.97 bcde	6.2 ± 0.85 ab	3.49 ± 0.17 a	3.6 ± 0.07 ab	4.6 ± 0.42 ab	3.53 ± 0.72 bcd	0.74 ± 0.03 ab	0.77 ± 0.06 abcd
‘Verdello Blanco’	VB	08/09/2015	25.7	257.7	4.7	3.77	2.4	7.7	5.47	1.64	0.31
		07/09/2016	26.0	266.5	5.7	3.33	1.7	7.0	4.56	1.23	0.24
		11/09/2017	23.3	229.7	6.7	3.25	2.5	6.5	3.48	0.97	0.38
			25.0 ± 1.48 e	251.3 ± 19.22 f	5.7 ± 1.00 ab	3.45 ± 0.28 a	2.2 ± 0.44 a	7.1 ± 0.60 cd	4.50 ± 1.00 d	1.28 ± 0.34 c	0.31 ± 0.07 a
Significance			***	***	*	ns	**	**	**	**	**

MI-CO: maturation index De Cillis and Odifredi. MI-BS: maturation index Baragiola and Scuppli. T:M: Relation tartaric:malic acid. *, **, *** and ns indicate significance at *p* ≤ 0.05, *p* ≤ 0.01, *p* ≤ 0.0001 and non-significant difference, respectively. Mean value, SD and different roman letters (a–e), showing significant differences according to Fisher’s test (*p* < 0.05), are indicated in bold for each variety.

**Table 2:** Free volatile compounds in white varieties in 2015, 2016 and 2017 vintages. (Values are expressed as µg·L^−1^).

**Varitey**	**Year**	**ACIDS**	**ALCOHOLS**	**ALDEHYDES**	**C6**	**ESTERS**	**PHENOLS**	**THIOLS**
AL	2015	1101.97	815.93	49.39	1966.07	n.d.	10.59	n.d.
	2016	271.96	708.13	69.83	1269.01	n.d.	6.56	8.00
	2017	46.01	713.94	30.23	1830.68	n.d.	n.d.	n.d.
		473.31 ± 556.03 ab	746.00 ± 60.63 a	49.82 ± 19.81 a	1688.58 ± 369.62 a	n.d. a	5.72 ± 5.35 a	2.67 ± 4.62 ab
BB	2015	109.28	817.04	199.25	991.98	n.d.	n.d.	3.95
	2016	147.40	783.40	17.26	3472.93	14.80	10.04	n.d.
		128.34 ± 26.96 a	800.22 ± 23.79 a	108.26 ± 128.69 a	2232.45 ± 1754.29 ab	7.40 ± 10.46 a	5.02 ± 7.10 a	1.97 ± 2.79 ab
CBL	2015	558.73	623.09	125.30	1597.37	n.d.	22.79	n.d.
	2016	197.47	709.80	23.93	423.52	179.89	4.46	n.d.
	2017	478.39	751.95	41.09	1895.42	n.d.	74.99	n.d.
		411.53 ± 189.68 a	694.95 ± 65.70 a	63.44 ± 54.26 a	1305.44 ± 778.16 a	59.96 ± 103.86 a	34.08 ± 36.60 a	n.d. ab
LO	2015	230.10	566.16	25.06	717.64	154.89	n.d.	n.d.
	2016	277.75	575.16	20.07	2192.41	n.d.	n.d.	n.d.
	2017	184.82	789.22	34.10	3009.24	n.d.	39.09	n.d.
		230.89 ± 46.47 a	643.51 ± 126.27 a	26.41 ± 7.11 a	1973.09 ± 1161.44 a	51.63 ± 89.43 a	13.03 ± 22.57 a	n.d. a
VB	2015	3978.75	3554.47	454.96	8097.34	n.d.	71.15	n.d.
	2016	766.55	782.70	62.23	2863.73	16.60	9.51	n.d.
	2017	527.99	508.63	34.60	1449.00	n.d.	n.d.	n.d.
		1757.76 ± 1927.13 b	1615.27 ± 1684.98 a	183.93 ± 235.12 ab	4136.69 ± 3502.20 ab	5.53 ± 9.58 a	26.89 ± 38.63 a	n.d. a
Significance		ns	ns	ns	ns	ns	ns	ns
**Varitey**	**Year**	**KETONES**	**PAHs**	**LACTONS**	**NORISOPRENOIDS**	**SESQUITERPENES**	**TERPENES**	**TOTAL**
AL	2015	113.94	62.02	n.d.	69.74	n.d.	712.35	4902.00
	2016	127.42	96.94	32.38	n.d.	n.d.	181.07	2771.30
	2017	92.71	103.69	n.d.	n.d.	n.d.	654.56	3471.80
		111.36 ± 17.50 abcd	87.55 ± 22.37 a	10.79 ± 18.67 ab	23.25 ± 40.26 ab	n.d. a	515.99 ± 291.49 a	3715.04 ± 1085.97 ab
BB	2015	47.19	99.20	23.74	n.d.	n.d.	133.57	2425.22
	2016	53.32	48.26	27.24	19.85	n.d.	91.35	4685.85
		50.26 ± 4.33 a	73.73 ± 36.02 a	25.49 ± 2.48 ab	9.93 ± 14.04 ab	n.d. a	112.46 ± 29.86 a	3555.53 ± 1598.51 ab
CBL	2015	279.19	176.34	58.64	83.86	n.d.	74.49	3599.77
	2016	80.25	89.98	42.98	5.64	n.d.	26.54	1784.45
	2017	93.15	40.86	50.65	60.24	n.d.	70.37	3557.11
		150.86 ± 111.32 abcd	102.39 ± 68.59 a	50.75 ± 7.83 ab	49.91 ± 40.12 b	n.d. a	57.13 ± 26.57 a	2980.44 ± 1035.98 a
LO	2015	9.85	10.72	1.08	9.66	n.d.	210.12	1935.29
	2016	161.78	120.57	105.43	n.d.	n.d.	1046.87	4500.04
	2017	93.00	95.29	41.14	11.81	n.d.	3818.06	8115.77
		88.21 ± 76.08 ab	75.53 ± 57.53 a	49.22 ± 52.64 ab	7.15 ± 6.29 a	n.d. a	1691.69 ± 1888.43 b	4850.37 ± 3105.10 ab
VB	2015	147.76	75.95	n.d.	5.87	n.d.	467.29	16,853.53
	2016	41.12	91.93	4.51	12.55	n.d.	498.63	5150.06
	2017	92.02	57.37	n.d.	32.26	n.d.	785.37	3487.24
		93.64 ± 53.34 abc	75.08 ± 17.30 a	1.50 ± 2.60 a	16.89 ± 13.72 ab	n.d. a	583.76 ± 175.30 a	8496.94 ± 7284.62 ab
Significance		ns	ns	ns	ns	ns	ns	ns

PAHs.: polycyclic aromatic hydrocarbons; AL: ‘Albariño’; BB: ‘Brancellao Blanco’; CBL: ‘Caíño Blanco’; LO: ‘Loureira’; VB: ‘Verdello Blanco’; n.d.: none detected. ns indicates non-significant difference. Internal standards: 4 metil-2-pentanol for C6 compounds, aldehydes, acids, esters, alcohols and thiols. 3-octanol for terpenes, ketones, aromatic hydrocarbons, lactones, norisoprenoids and sesquiterpenes. Mean value, SD and different roman letters (a–d), showing significant differences according to Fisher’s test (*p* < 0.05), are indicated in bold for each variety.

**Table 3:** Free volatile compounds in red varieties in 2015, 2016 and 2017 vintages. (Values are expressed as µg·L^−1^).

**Varitey**	**Year**	**ACIDS**	**ALCOHOLS**	**ALDEHYDES**	**C6**	**ESTERS**	**PHENOLS**	**THIOLS**
BR	2015	2489.22	2271.32	1571.65	9718.94	34.14	n.d.	n.d.
	2016	189.72	768.28	192.00	3881.60	15.57	n.d.	n.d.
	2017	412.87	1077.82	124.38	4026.64	n.d.	127.84	n.d.
		1030.60 ± 1268.11 ab	1372.48 ± 793.66 a	629.34 ± 816.76 b	5875.73 ± 3329.11 b	16.57 ± 17.09 a	42.61 ± 73.81 a	n.d. a
CB	2015	967.74	852.98	74.05	1563.81	n.d.	16.54	n.d.
	2016	445.56	743.40	13.77	1929.67	8.80	11.05	n.d.
	2017	308.00	871.41	72.95	1488.25	n.d.	8.31	n.d.
		573.77 ± 348.06 ab	822.60 ± 69.20 a	53.59 ± 34.4 a	1660.58 ± 236.08 a	2.93 ± 5.08 a	11.96 ± 4.19 a	n.d. a
CL1	2015	1349.73	808.82	22.85	1821.20	n.d.	13.95	n.d.
	2016	n.d.	782.90	171.75	8422.85	18.37	4.74	n.d.
	2017	368.45	741.63	n.d.	1605.88	4.29	16.41	n.d.
		572.73 ± 697.66 ab	777.78 ± 33.88 a	64.87 ± 93.27 a	3949.98 ± 3875.12 ab	7.55 ± 9.61 a	11.70 ± 6.15 a	n.d. a
CL2	2015	488.02	532.94	19.66	1267.26	n.d.	11.76	n.d.
	2016	219.19	809.16	57.27	4730.51	22.38	8.30	n.d.
	2017	194.08	1211.98	31.26	3963.27	n.d.	57.39	n.d.
		300.43 ± 162.94 a	851.36 ± 341.48 a	36.06 ± 19.26 a	3320.35 ± 1818.94 ab	7.46 ± 12.92 a	25.82 ± 27.40 a	n.d. a
CT	2015	444.61	440.89	64.64	971.31	n.d.	8.64	n.d.
	2016	212.44	758.20	267.82	5276.87	10.66	n.d.	n.d.
	2017	446.06	755.78	n.d.	2976.84	n.d.	41.60	n.d.
		367.70 ± 134.46 a	651.62 ± 182.51 a	110.82 ± 139.75 a	3075.01 ± 2154.46 ab	3.55 ± 6.15 a	16.75 ± 21.95 a	n.d. a
CS	2015	727.01	487.21	35.22	1912.33	n.d.	17.02	70.06
	2016	447.00	670.23	26.33	2422.25	n.d.	19.01	n.d.
	2017	376.56	882.76	58.66	2753.54	7.62	78.93	n.d.
		516.86 ± 185.37 ab	680.07 ± 197.96 a	40.07 ± 16.70 a	2362.71 ± 423.75 ab	2.54 ± 4.40 a	38.32 ± 35.18 a	23.35 ± 40.45 b
SO	2016	273.54	1008.33	74.32	4489.44	60.19	n.d.	8.07
	2017	417.49	803.20	30.60	1383.96	n.d.	7.74	n.d.
		345.52 ± 101.79 a	905.77 ± 145.05 a	52.46 ± 30.92 a	2936.70 ± 2195.91 ab	30.09 ± 42.56 a	3.87 ± 5.47 a	4.03 ± 5.70 ab
Significance		ns	ns	ns	ns	ns	ns	ns
**Varitety**	**Year**	**KETONES**	**PAHs**	**LACTONS**	**NORISOPRENOIDS**	**SESQUITERPENES**	**TERPENES**	**TOTAL**
BR	2015	145.21	47.07	39.24	n.d.	n.d.	208.34	16,525.12
	2016	109.27	92.32	63.50	n.d.	n.d.	13.71	5325.98
	2017	198.04	109.94	n.d.	n.d.	n.d.	457.68	6535.21
		150.84 ± 44.65 abcd	83.11 ± 32.43 a	34.24 ± 32.04 ab	n.d. a	n.d. a	226.57 ± 222.54 a	9462.10 ± 6146.56 b
CB	2015	170.96	87.23	49.40	17.84	n.d.	48.99	3849.54
	2016	164.89	118.71	59.38	n.d.	n.d.	14.08	3509.32
	2017	148.73	98.70	8.19	3.04	n.d.	43.73	3051.32
		161.53 ± 11.49 bcd	101.55 ± 15.93 a	38.99 ± 27.14 ab	6.96 ± 9.54 a	n.d. a	35.60 ± 18.82 a	3470.06 ± 400.554 a
CL1	2015	229.98	97.57	72.42	32.41	n.d.	63.13	4512.05
	2016	171.33	83.69	129.82	n.d.	n.d.	23.45	9808.91
	2017	139.33	62.75	6.01	18.20	n.d.	123.20	3086.15
		180.21 ± 45.97 bcd	81.34 ±17.53 a	69.42 ± 61.96 ab	16.87 ± 16.25 ab	n.d. a	69.92 ± 50.22 a	5802.37 ± 3542.26 ab
CL2	2015	178.70	98.62	5.64	52.71	n.d.	511.76	3167.08
	2016	133.03	72.40	27.83	n.d.	n.d.	82.34	6162.40
	2017	265.83	52.73	211.13	n.d.	n.d.	177.17	6164.84
		192.52 ± 67.47 d	74.58 ± 23.03 a	81.53 ± 112.78 b	17.57 ± 30.43 ab	n.d. a	257.09 ± 225.60 a	5164.77 ± 1730.05 ab
CT	2015	156.93	55.49	31.97	27.69	n.d.	5.98	2208.16
	2016	70.32	73.75	74.33	n.d.	n.d.	11.69	6756.07
	2017	170.01	96.73	53.04	22.47	2.25	36.49	4601.28
		132.42 ± 54.17 abcd	75.33 ± 20.66 a	53.11 ± 21.18 ab	16.72 ± 14.71 ab	0.75 ± 1.30 b	18.05 ± 16.22 a	4521.84 ± 2275.00 ab
CS	2015	229.81	191.49	55.44	3.85	n.d.	7.76	3737.20
	2016	167.63	79.99	62.50	n.d.	n.d.	9.27	3904.22
	2017	166.96	91.23	93.58	11.05	n.d.	21.80	4542.69
		188.13 ± 36.09 cd	120.90 ± 61.39 a	70.51 ± 20.29 ab	4.96 ± 5.61 a	n.d. a	12.95 ± 7.70 a	4061.37 ± 425.12 ab
SO	2016	127.32	81.65	46.84	n.d.	n.d.	18.70	6188.39
	2017	171.08	37.72	27.22	6.05	n.d.	5.07	2890.13
		149.20 ± 30.95 abcd	59.68 ± 31.07 a	37.03 ± 13.87 ab	3.02 ± 4.28 a	n.d. a	11.89 ± 9.63 a	4539.26 ± 2332.22 ab
Significance		ns	ns	ns	ns	ns	ns	ns

PAHs.: polycyclic aromatic hydrocarbons; BR: ‘Brancellao’; CB: ‘Caíño Bravo’; CL1: ‘Caíño Longo 1’; CL2: ‘Caíño Longo 2’; CT: ‘Caíño Tinto’; CS: ‘Castañal’; SO: ‘Sousón’; n.d.: none detected. ns indicates non-significant difference. Internal standards: 4 metil-2-pentanol for C6 compounds, aldehydes, acids, esters, alcohols and thiols. 3-octanol for terpenes, ketones, aromatic hydrocarbons, lactones, norisoprenoids and sesquiterpenes. Mean value, SD and different roman letters (a–d), showing significant differences according to Fisher’s test (*p* < 0.05), are indicated in bold for each variety.

**Table 4:** Aromatic precursors in white varieties in 2015, 2016 and 2017 vintages. (Values are expressed as µg·L^−1^).

**Varitey**	**Year**	**ACIDS**	**ALCOHOLS**	**ALDEHYDES**	**C6**	**ESTERS**	**PHENOLS**	**THIOLS**
AL	2015	3019.63	9891.19	n.d.	1338.76	3033.73	1377.61	n.d.
	2016	2010.49	9585.60	n.d.	1641.33	30,203.90	35,100.21	n.d.
	2017	4081.37	7856.00	n.d.	807.50	9308.53	627.17	33.69
		3037.16 ± 1035.55 c	9110.93 ± 1097.48 ab	n.d. a	1262.53 ± 422.11 a	14,182.05 ± 14,225.61 ab	12,368.33 ± 19,689.96 a	11.23 ± 19.45 a
BB	2015	n.d.	16,978.93	n.d.	4482.51	461.88	4905.18	151.39
	2016	565.41	16,342.97	n.d.	3379.83	3915.19	31,755.56	123.60
		282.70± 399.80 a	16,660.95 ± 449.70 ab	n.d. a	3931.17 ± 779.71 abc	2188.54 ± 2441.86 ab	18,330.37 ± 18,986.09 ab	137.49 ± 19.65 b
CBL	2015	1864.69	9015.35	307.84	1289.48	2940.50	1604.07	41.55
	2016	750.43	18,765.05	614.13	1284.78	2295.65	1143.00	n.d.
	2017	187.85	14,905.96	634.53	892.51	2918.33	653.31	n.d.
		934.33 ± 853.41 abc	14,228.79 ± 4910.00 ab	518.83 ± 183.01 ab	1155.59 ± 227.84 a	2718.16 ± 366.07 ab	1133.46 ± 475.45 a	13.85 ± 23.99 a
LOok	2015	2519.85	30,128.66	n.d.	2770.64	4510.74	780.03	208.36
	2016	5923.58	14,015.65	927.05	742.75	6556.50	2870.47	n.d.
	2017	311.25	19,506.57	n.d.	1006.72	1123.28	1546.78	32.30
		2918.23 ± 2827.29 bc	21,216.96 ± 8191.54 ab	309.02 ± 535.23 a	1506.70 ± 1102.53 ab	4063.50 ± 2744.08 ab	1732.43 ± 1057.52 a	80.22 ± 112.14 ab
VB	2015	298.09	20,335.81	n.d.	2448.60	1093.66	13,129.63	n.d.
	2016	706.56	14,381.42	n.d.	4003.46	3948.07	11,603.12	n.d.
	2017	582.13	7419.42	n.d.	998.07	1623.99	7883.99	n.d.
		528.93 ± 209.37 abc	14,045.55 ± 6464.74 ab	n.d. a	2483.38 ± 1503.00 ab	2221.91 ± 1518.24 ab	10,872.25 ± 2698.11 a	n.d. a
Significance		ns	ns	ns	ns	ns	ns	ns
**Varitey**	**Year**	**KETONES**	**PAHs**	**LACTONS**	**NORISOPRENOIDS**	**SESQUITERPENES**	**TERPENES**	**TOTAL**
AL	2015	118.48	32.88	n.d.	64.78	n.d.	8823.94	27,701.00
	2016	341.65	59.50	n.d.	n.d.	5.13	4661.78	83,609.59
	2017	143.86	126.37	n.d.	n.d.	n.d.	6690.97	29,675.46
		201.33 ± 122.18 a	72.92 ± 48.17 a	n.d.	21.59 ± 37.40 a	1.71 ± 2.96 a	6725.56 ± 2081.30 ab	46,995.35 ± 31,724.22 ab
BB	2015	149.46	72.17	n.d.	n.d.	6.20	4006.16	31,213.86
	2016	184.78	29.94	n.d.	n.d.	7.05	1622.41	57,926.73
		167.12 ± 24.98 a	51.05 ± 29.86 a	n.d.	n.d. a	6.62 ± 0.61 ab	2814.29 ± 1685.56 a	44,570.29 ± 18,888.9 ab
CBL	2015	118.94	8.86	n.d.	n.d.	6.81	1316.99	18,515.07
	2016	324.11	72.06	n.d.	n.d.	n.d.	466.76	25,715.97
	2017	168.69	79.07	n.d.	15.27	n.d.	2364.42	22,819.94
		203.91 ± 107.03 a	53.33 ± 38.67 a	n.d.	5.09 ± 8.82 a	2.27 ± 3.93 a	1382.72 ± 950.54 a	22,350.33 ± 3623.35 a
LO	2015	85.75	19.12	n.d.	n.d.	n.d.	46,750.84	87,773.99
	2016	748.14	181.36	n.d.	n.d.	n.d.	244.66	32,210.17
	2017	189.61	142.14	n.d.	27.53	n.d.	8688.19	32,574.36
		341.17 ± 356.26 a	114.21 ± 84.65 a	n.d.	9.18 ± 15.89 a	n.d. a	18,561.23 ± 24,775.27 b	50,852.84 ± 31,975.2 ab
VB	2015	30.51	32.90	n.d.	n.d.	23.38	20,038.14	57,430.71
	2016	128.36	46.07	n.d.	n.d.	10.65	5672.47	40,500.18
	2017	135.99	52.02	n.d.	n.d.	12.03	5816.19	24,523.85
		98.29 ± 58.82 a	43.66 ± 9.79 a	n.d.	n.d. a	15.35 ± 6.98 b	10,508.93 ± 8252.85 ab	40,818.24 ± 16,455.7 a
Significance		ns	ns	ns	ns	*	ns	ns

PAHs.: polycyclic aromatic hydrocarbons; AL: ‘Albariño’; BB: ‘Brancellao Blanco’; CBL: ‘Caíño Blanco’; LO: ‘Loureira’; VB: ‘Verdello Blanco’; n.d.: none detected. * and ns indicate significance at *p* ≤ 0.05 and non-significant difference, respectively. Internal standards: 4 metil-2-pentanol for C6 compounds, aldehydes, acids, esters, alcohols and thiols. 3-octanol for terpenes, ketones, aromatic hydrocarbons, lactones, norisoprenoids and sesquiterpenes. Mean value, SD and different roman letters (a–c) showing significant differences according to Fisher’s test (*p* < 0.05), are indicated in bold for each variety.

**Table 5:** Aromatic precursors in red varieties in 2015, 2016 and 2017 vintages. (Values are expressed as µg·L^−1^).

**Varitey**	**Year**	**ACIDS**	**ALCOHOLS**	**ALDEHYDES**	**C6**	**ESTERS**	**PHENOLS**	**THIOLS**
BR	2016	52.13	7589.07	905.29	2779.75	811.05	375.78	n.d.
	2017	648.89	8665.05	96.79	2532.82	3376.00	624.24	51.01
		350.51 ± 421.97 ab	8127.06 ± 760.83 a	501.04 ± 571.70 ab	2656.29 ± 174.60 ab	2093.53 ± 1813.70 ab	500.01 ± 175.69 a	25.50 ± 36.07 ab
CB	2015	n.d.	2552.86	111.82	1228.72	297.24	43.82	6.18
	2016	n.d.	7104.75	1147.83	1537.91	876.21	742.53	n.d.
	2017	45.25	16,600.43	1550.90	2798.26	10,955.88	1066.82	187.09
		15.08 ± 26.12 a	8752.68 ± 7167.31 a	936.85 ± 742.37 b	1854.96 ± 831.41 a	4043.11 ± 5993.63 ab	617.72 ± 522.79 a	64.42 ± 106.27 ab
CL1	2015	48.10	12,566.99	28.04	5440.30	4181.46	406.55	n.d.
	2016	3095.84	17,794.79	991.25	3268.05	3664.16	1277.28	n.d.
	2017	605.88	12,571.19	403.39	2635.03	6822.39	529.28	72.39
		1249.94 ± 1622.74 abc	14,310.99 ± 3017.06 ab	474.23 ± 485.49 ab	3781.13 ± 1471.33 abc	4889.34 ± 1693.94 ab	737.70 ± 471.30 a	24.13 ± 41.79 a
CL2	2015	741.40	31,355.27	1462.81	3580.88	4097.04	527.16	38.84
	2016	1140.05	11,292.29	1024.03	1586.45	48,309.24	59,369.35	n.d.
	2017	2141.12	10,201.90	n.d.	1038.33	2891.92	39,573.59	n.d.
		1340.86 ± 721.14 abc	17,616.48 ± 11,910.62 ab	828.95 ± 750.66 b	2068.56 ± 1338.08 ab	18,432.73 ± 25,880.83 b	33,156.70 ± 29,941.33 b	12.95 ± 22.42 a
CT	2015	550.10	16,136.63	575.33	6243.90	3027.39	277.67	14.63
	2016	n.d.	8737.97	726.66	1970.69	652.70	241.68	n.d.
	2017	515.04	9779.40	480.84	1818.47	2584.03	220.58	58.00
		355.05 ± 307.98 b	11,551.33 ± 4004.98 ab	594.28 ± 124. 00 ab	3344.35 ± 2512.23 abc	2088.04 ± 1262.65 ab	246.64 ± 28.87 a	24.21 ± 30.16 a
CS	2015	170.97	7616.96	366.75	3340.49	128.60	3042.85	8.54
	2016	5393.14	26,681.64	1138.59	9393.17	618.44	1888.44	n.d.
	2017	194.78	5887.93	355.37	2098.40	646.70	1422.14	34.84
		1919.63 ± 3008.17 abc	13,395.51 ± 11,538.56 ab	620.24 ± 448.94 ab	4944.02 ± 3902.81 bc	464.58 ± 291.31 a	2117.81 ± 834.35 a	14.46 ± 18.16 a
SO	2016	n.d.	7607.23	659.33	5273.31	694.38	934.71	145.72
	2017	239.98	9657.28	498.21	7488.80	5042.70	357.77	10.65
		119.99 ± 169.69 a	8632.26 ± 1449.60 a	578.77 ± 113.93 ab	6381.05 ± 1566.59 c	2868.54 ± 3074.73 ab	646.24 ± 407.96 a	78.18 ± 95.51 ab
Significance		ns	ns	ns	ns	ns	ns	ns
**Variety**	**Year**	**KETONES**	**PAHs**	**LACTONS**	**NORISOPRENOIDS**	**SESQUITERPENES**	**TERPENES**	**TOTAL**
BR	2016	334.24	49.58	n.d.	n.d.	n.d.	125.60	13,022.50
	2017	174.44	93.23	n.d.	n.d.	8.97	3105.49	19,376.94
		254.34 ± 113.00 a	71.41 ± 30.87 a	n.d. a	n.d. a	4.48 ± 6.34 a	1615.54 ± 2107.10 a	16,199.72 ± 4493.27 a
CB	2015	38.63	n.d.	n.d.	n.d.	n.d.	3215.81	7495.09
	2016	513.96	102.00	n.d.	n.d.	n.d.	79.48	12,104.67
	2017	180.71	91.80	n.d.	n.d.	n.d.	2973.04	36,450.17
		244.44 ± 243.99 a	64.60 ± 56.18 a	n.d. a	n.d. a	n.d. a	2089.44 ± 1744.91 a	18,683.31 ± 15,558.21 a
CL1	2015	11.68	n.d.	n.d.	n.d.	n.d.	644.27	23,327.39
	2016	671.44	106.94	n.d.	n.d.	n.d.	167.29	31,037.03
	2017	197.65	81.46	n.d.	n.d.	n.d.	2846.66	26,765.32
		293.59 ± 340.18 a	62.80 ± 55.86 a	n.d. a	n.d.	n.d.	1219.40 ± 1429.28 a	27,043.25 ± 3862.33 a
CL2	2015	38.52	n.d.	13.66	4.51	n.d.	9770.69	51,671.34
	2016	323.55	30.10	n.d.	n.d.	n.d.	5212.03	128,287.10
	2017	202.18	n.d.	n.d.	n.d.	23.48	6177.23	62,249.75
		188.08 ± 143.03 a	10.03 ± 17.38 a	4.55 ± 7.88 b	1.50 ± 2.60 a	7.83 ± 13.56 ab	7053.32 ± 2402.29 ab	80,736.06 ± 41,518.69 b
CT	2015	47.69	n.d.	n.d.	n.d.	n.d.	587.32	27,460.67
	2016	220.49	134.16	n.d.	n.d.	n.d.	371.38	13,055.71
	2017	204.85	73.96	n.d.	n.d.	n.d.	718.32	16,453.49
		157.68 ± 95.57 a	69.37 ± 67.20 a	n.d. a	n.d. a	n.d. a	559.01 ± 175.20 a	18,989.96 ± 7530.00 a
CS	2015	171.71	28.26	n.d.	n.d.	n.d.	1175.16	16,050.28
	2016	180.43	255.66	n.d.	n.d.	n.d.	59.02	45,608.52
	2017	110.41	32.75	n.d.	n.d.	n.d.	1336.09	12,119.40
		154.18 ± 38.16 a	105.56 ± 130.01 a	n.d. a	n.d. a	n.d. a	856.76 ± 695.53 a	24,592.73 ± 18,306.02 a
SO	2016	233.70	175.85	n.d.	n.d.	n.d.	442.19	16,166.43
	2017	140.49	61.92	n.d.	n.d.	n.d.	1830.86	25,328.66
		187.10 ± 65.91 a	118.88 ± 80.57 a	n.d. a	n.d. a	n.d. a	1136.53 ± 981.93 a	20,747.55 ± 6478.67 a
Significance		ns	ns	ns	ns	*	ns	ns

PAHs.: polycyclic aromatic hydrocarbons; BR: ‘Brancellao’; CB: ‘Caíño Bravo’; CL1: ‘Caíño Longo 1’; CL2: ‘Caíño Longo 2’; CT: ‘Caíño Tinto’; CS: ‘Castañal’; SO: ‘Sousón’; n.d.: none detected. * and ns indicate significance at *p* ≤ 0.05 and non-significant difference, respectively. Internal standards: 4 metil-2-pentanol for C6 compounds, aldehydes, acids, esters, alcohols and thiols. 3-octanol for terpenes, ketones, aromatic hydrocarbons, lactones, norisoprenoids and sesquiterpenes. Mean value, SD and different roman letters (a–c), showing significant differences according to Fisher’s test (*p* < 0.05), are indicated in bold for each variety.

**Table 6:** Major volatile compounds in white varieties in free and bound form.

Compound	Family	AL	BB	CBL	LO	VB
F	Acids	Hexanoic acid			Nonanoic acid	Hexanoic acid
F	Alcohols	1-Hexanol, 2-ethyl-				
F	C6	2-Hexenal, (E)-				
F	Aldehydes	(E)-2-Nonenal	Benzaldehyde, 2,5-dimethyl-	(E)-2-Nonenal	cis-2-Nonenal	Benzaldehyde, 2,5-dimethyl-
F	Esters		Hexanoic acid, phenethyl ester	Octanoic acid, ethyl ester	Butanoic acid, 3-methyl-, 2-methylbutyl ester	Hexanoic acid, ethyl ester
F	Phenols	Estragole		Phenol, 2,6-bis(1,1-dimethylethyl)-4-methyl-		Estragole
F	Ketones	Sulcatone			Cyclohexanone, 2-methyl-	Sulcatone
F	PAHs	Hemimellitene	psi.-Cumene	Hemimellitene		
F	Lactons	Tetrahydrofuran-2-one,4,4,5,5-tetramethyl			2,5-Dimethyl-4-hydroxy-3(2H)-furanone	2(3H)-Benzofuranone, hexahydro-3-methylene-
F	Norisoprenoids	α-Damascenone	δ-Damascone	α-Damascenone		
F	Sesquiterpenes					
F	Terpenes	α-Terpineol	Nerol	α-Terpineol	Lynalil antranilato	Linalool
F	Thiols	Nonanal, 3-(methylthio)-	2-Undecanethiol, 2-methyl-			
A.P.	Acids	Nonanoic acid		Octanoic Acid		
A.P.	Alcohols	Benzyl alcohol		2phenylethanol		
A.P.	C6	1-Hexanol				
A.P.	Aldehydes			Benzaldehyde		
A.P.	Esters	Nonanoato de etilo	Nonanoic acid, methyl ester	Methyl Salicylate	Octanoic acid, methyl ester	Methyl Salicylate
A.P.	Phenols	Phenol, 2,4-di-tert-butyl-	Phenol, 4-ethyl-	Phenol, 2,4-di-tert-butyl-		Phenol, 4-ethyl-
A.P.	Ketones	Methanone, diphenyl-	3-Octanone	Acetophenone		3-Octanone
A.P.	PAHs	Mesitylene	psi.-Cumene		Hemimellitene	psi.-Cumene
A.P.	Lactons					
A.P.	Norisoprenoids	α-Damascenone		Dihydro-β-ionol		
A.P.	Sesquiterpenes	Patchoulane				Patchoulane
A.P.	Terpenes	Linalool	3-Octanol, 3,6-dimethyl-	α-Terpineol	Linalool	
A.P.	Thiols	2-Undecanethiol, 2-methyl-				

F: free volatile; A.P.: aromatic precursors; PAH’s: polycyclic aromatic hydrocarbons; AL: ‘Albariño’; BB: ‘Brancellao’; CBL: ‘Caíño Blanco’; LO: ‘Loureira’; VB: ‘Verdello Blanco’.

**Table 7:** Major volatile compounds in red varieties in free and bound form.

Compound	Family	BR	CB	CL1	CL2	CT	CS	SO
F	Acids	Nonanoic acid			Hexanoic acid		Nonanoic acid	Hexanoic acid
F	Alcohols	1-Hexanol, 2-ethyl-						
F	C6	2-Hexenal, (E)-	Hexanal		2-Hexenal, (E)-		1-Hexanol	
F	Aldehydes	Benzaldehyde, 2,5-dimethyl-	cis-4-Decenal	Benzaldehyde	cis-2-Nonenal	Acetaldehyde, phenyl-	Benzaldehyde, 2,5-dimethyl-	Benzaldehyde
F	Esters	E-2-Hexenyl benzoate	Hexanoic acid, phenethyl ester	Hexanoic acid, ethyl ester		Hexanoic acid, phenethyl ester	Hexanoic acid, ethyl ester	Hexanoic acid, phenethyl ester
F	Phenols	Phenol, 2,6-bis(1,1-dimethylethyl)-4-methyl-	Estragole		Phenol, 2,6-bis(1,1-dimethylethyl)-4-methyl-			
F	Ketones	Sulcatone	2-Hexanone, 4-methyl-	Cyclohexanone, 2-methyl-	3,5-di-tert-Butyl-4-hydroxyacetophenone	4,5-Dihydro-2-methylimidazole-4-one	Cyclopentanone, 2-(1-methylpropyl)-	α-Methylcyclopentanone
F	PAHs	Mesitylene		Hemimellitene			psi.-Cumene	Hemimellitene
F	Lactons	Tetrahydrofuran-2-one,4,4,5,5-tetramethyl			Butyrolactone	Tetrahydrofuran-2-one,4,4,5,5-tetramethyl		
F	Norisoprenoids		α-Damascenone					β-Damascenone
F	Sesquiterpenes					α-Calacorene		
F	Terpenes	Lynalil antranilate	Nerol	α-Citronellol	α-Terpineol	Terpinil acetate		
F	Thiols						Nonanal, 3-(methylthio)-	
A.P.	Acids	Nonanoic acid	n-Octanoic acid	Octanoic Acid	Nonanoic acid		Decanoic acid	Nonanoic acid
A.P.	Alcohols	Benzyl alcohol		2-phenylethanol		Benzyl alcohol	2-phenylethanol	
A.P.	C6	1-Hexanol						
A.P.	Aldehydes	Benzaldehyde						
A.P.	Esters	Methyl Salicylate			Nonanoic acid, methyl ester	Methyl Salicylate	Octanoic acid, methyl ester	Methyl Salicylate
A.P.	Phenols	Dihydroeugenol		Phenol, 2,4-di-tert-butyl-				
A.P.	Ketones	Acetophenone			Methanone, diphenyl-	3-Heptanone, 5-methyl-	Methanone, diphenyl-	Acetophenone
A.P.	PAHs	psi.-Cumene	Mesitylene	psi.-Cumene		Mesitylene	Hemimellitene	Mesitylene
A.P.	Lactons				γ-Undecanolactone			
A.P.	Norisoprenoids				α-Damascenone			
A.P.	Sesquiterpenes	Patchoulane			Ledene oxide-(II)			
A.P.	Terpenes	Thymol			Linalool	α-Terpineol	Nerol	Thymol
A.P.	Thiols	2-Undecanethiol, 2-methyl-						

F: free volatile; A.P.: aromatic precursors; PAH’s: polycyclic aromatic hydrocarbons; BR: ‘Brancellao’; CB: ‘Caíño Bravo’; CL1: ‘Caíño Longo 1’; CL2: ‘Caíño Longo 2’; CT: ‘Caíño Tinto’; CS: ‘Castañal’; SO: ‘Sousón’.

## Data Availability

Data is contained within the article or supplementary material.

## Supplementary Materials

The following supporting information can be downloaded at: https://www.mdpi.com/article/10.3390/foods11101427/s1, Table S1: Yearly (2015, 2016 and 2017) bioclimatic indices and climatic data in the experimental plot site in northwest Spain (EVEGA, Ourense); Tables S2 and S3: Free aromatic fraction profile (Values are expressed as percentages); Tables S4 and S5 Aromatic precursor fraction profile (Values are expressed as percentages).

## References

[1] Díaz-Losada E., Salgado A.T., Orriols-Fernández I., Ramos-Cabrer A.M., Pereira-Lorenzo S. (2013). New synonyms and homonyms for cultivars from northwestern Spain. Am. J. Enol. Viticult..

[2] García-Muñoz S., Asproudi A., Cabello F., Borsa D. (2011). Aromatic characterization and enological potential of 21 minor varieties (*Vitis vinifera* L.). Eur. Food Res. Technol..

[3] Gutiérrez-Gamboa G., Liu S.Y., Pszczolkowski P. (2019). Resurgence of minority and autochthonous grapevine varieties in South America: A review of their oenological potential. J. Sci. Food Agric..

[4] Augusto D., Oliveira A.A., Falco V., Castro I. (2019). Uncovering Northeast Portugal grapevine´s varietal legacy. Vitis J. Grapevine Res..

[5] Lacombe T. These: Contribution á l´Étude de l´Histoire Evolutive de la Vigne Cultivée (*Vitis vinifera* L.) par l´Analyse de la Diversité Génétique Neuter et de Genes d´Intérêt. Ph.D. Dissertation.

[6] Organisation of Vine and Wine 2017 Report on the World Vitivinicultural Situation. https://www.oiv.int/en/oiv-life/oiv-2017-report-on-the-world-vitivinicultural-situation.

[7] Loureiro M.D., Moreno-Sanz P., Suárez B. (2017). Agronomical characterization of minority grapevine cultivars from Asturias (Spain). Cienc. Tec. Vitivinic..

[8] Duchêne E. (2016). How can grapevine genetics contribute to the adaptation to climate change?. OENO One.

[9] Wolkovich E.M., García de Cortázar-Atauri I., Morales-Castilla I., Nicholas K.A., Lacombe T. (2018). From Pinot to Xinomavro in the world´s future wine-growing regions. Nat. Clim. Chang..

[10] Wood S.A., Karp D.S., De Clerck F., Kremen C., Naeem S., Palm C.A. (2015). Functional traits in agriculture: Agrobiodiversity and ecosystem services. Trends Ecol. Evol..

[11] Balda P., Sancha J.C., García J., Zheng W., de Martínez Toda F. (2016). Caracterización agronómica y enológica de las variedades tintas minoritarias de la D.O.Ca. Rioja. Enoviticultura.

[12] López R., Ezpeleta E., Sánchez I., Cacho J., Ferreira V. (2004). Analysis of the aroma intensities of volatile compounds released from mild acid hydrolysates of odourless precursors extracted from Tempranillo and Grenache grapes using gas chromatography-olfactometry. Food Chem..

[13] Alcalde-Eon C., García-Estévez I., Martín-Baz A., Rivas-Gonzalo J.C., Escribano-Bailón M.T. (2014). Anthocyanin and flavonol profiles of *Vitis vinifera* L. cv Rufete grapes. Biochem. Syst. Ecol..

[14] Álvarez-Casas M., Pajaro M., Lores M., García-Jares C. (2016). Polyphenolic composition and antioxidant activity of Galician monovarietal wines from native and experimental non-native white grape varieties. Int. J. Food Prop..

[15] Costa E., Cosme F., Jordão A.M., Mendes-Faia A. (2014). Anthocyanin profile and antioxidant activity from 24 grape varieties cultivated in two portuguese wine regions. J. Int. Sci. Vigne Vin..

[16] Díaz-Losada E., Salgado A.T., Ramos-Cabrer A.M., Río Segade S., Diéguez S.C., Pereira-Lorenzo S. (2010). Twenty microsatellites (SSRs) reveal two main origins of variability in grapevine cultivars from Northwestern Spain. Vitis.

[17] Díaz-Losada E., Salgado A.T., Ramos-Cabrer A.M., Pereira-Lorenzo S. (2011). Determination of genetic relationships of Albariño and Loureira cultivars with the Caíño group by microsatellites. Am. J. Enol. Viticult..

[18] Díaz-Losada E., Salgado A.T., Ramos-Cabrer A.M., Díaz-Hernández B., Pereira-Lorenzo S. (2012). Genetic and geographical structure in grapevines from northwestern Spain. Ann. Appl. Biol..

[19] Alleweldt G. (1988). The Genetic Resources of Vitis: Genetic and Geographic Origin of Cultivars, Their Prime Names, and Synonyms.

[20] Castro I., Martin J.P., Ortiz J.M., Pinto-Carnide O. (2011). Varietal discrimination and genetic relationships of *Vitis vinifera* L. cultivars from two major Controlled Appellation (DOC) regions in Portugal. Sci. Hortic..

[21] Cunha J., Ibáñez J., Teixeira-Santos M., Brazão J., Fevereiro P., Martínez-Zapater J.M., Eiras-Dias J.E. (2020). Genetic relationships among Portuguese cultivated and wild *Vitis vinifera* L. germplasm. Front. Plant Sci..

[22] Carpetieri A., Sebastianelli A., Melchiorre C., Pinto G., Trifuoggi M., Lettera V., Amoresano A. (2019). Fiano, Greco and Falanghina grape cultivars differentiation by volatiles fingerprinting, a case study. Heliyon.

[23] Conde C., Silva P., Fontes N., Dias A.C.P., Tavares R.M., Sousa M.J., Agasse A., Delrot S., Gerós H. (2007). Biochemical changes throughout grape berry development and fruit and wine quality. Food Spec. Feature.

[24] Oliveira J.M., Faria M., Sá F., Barros F., Araújo I.M. (2006). C6-alcohols as varietal markers for assessment of wine origin. Anal. Chim. Acta.

[25] Ribéreau-Gayon P., Glories Y., Maujean A., Dubourdieu D. (2006). Varietal aroma. Handbook of Enology. The Chemistry of Wine Stabilization and Treatments.

[26] Cabrita M.J., Freitas A.M.C., Laureano O., Di Stefano R. (2006). Glycosidic aroma compounds of some Portuguese grape cultivars. J. Sci. Food Agric..

[27] Antalick G., Šuklje K., Blackman J.W., Meeks C., Deloire A., Schmidtke L.M. (2015). Influence of grape composition on red wine ester profile: Comparison between Cabernet Sauvignon and Shiraz cultivars from Australian warm climate. J. Agric. Food Chem..

[28] Marais J. (1983). Terpenes in the aroma of grapes and wines: A review. S. Afr. J. Enol. Vitic..

[29] Rambla J.L., Trapero-Mozos A., Diretto G., Rubio-Moraga A., Granell A., Gómez-Gómez L., Ahrazem O. (2016). Gene-metabolite networks of volatile metabolism in Airen and Tempranillo grape cultivars revealed a distinct mechanism of aroma bouquet production. Front. Plant Sci..

[30] Huglin P. (1978). Nouveau mode d´évaluation des possibilités héliothermiques d´en milieu viticole. Comptes Rendus Acad. Agric. Fr..

[31] Organisation of Vine and Wine (2020). Compendium of International Methods of Wine and Must Analysis. Ed. https://www.oiv.int/public/medias/7372/oiv-compendium-volume-1-2020.pdf.

[32] Perestrelo R., Barros A.S., Rocha SMCamara J.S. (2014). Establishement of the varietal profile of *Vitis vinifera* L. grape varieties from different geographical regions based on HS-SPME/GC-qMS combined with chemometric tools. Michrochem. J..

[33] Di Stefano R. (1991). Proposition d’une méthode de preparation d’echantillon pour la détermination des terpenes libres et glycosides des raisins et des vins. Bull OIV.

[34] Diéguez S.C., Lois L.C., Gómez E.F., de la Peña M.L.G. (2003). Aromatic composition of the Vitis vinífera grape Albariño. Lebnsm.-Wiss. U.-Tecnol..

[35] Tonietto J., Carbonneau A. (2004). A multicriteria climatic classification system for grape-growing regions worldwide. Agric. For. Meteorol..

[36] Vilanova M., Cortés S., Santiago J.L., Martínez C., Fernández E. (2008). Contribution of some grape-derived aromatic compounds to the primary aroma in red wines from cv. Caiño Tinto, cv. Caiño Bravo and cv. Caiño Longo grapes. J. Agric. Sci..

[37] Tomasino E., Bolman S. (2021). The potential effect of β-Ionone and β-Damascenone on sensory perception of Pinot Noir wine aroma. Molecules.

[38] Slaghenaufi D., Peruch E., De Cosmi M., Nouvelet L., Ugliano M. (2021). Volatile and phenolic composition of monovarietal red wines of Valpolicella appellations. OENO One.

[39] The Good Scent Company. https://www.thegoodscentcompany.com.

[40] Ferreira V., Ortín N., Escudero A., López R., Cacho J. (2002). Chemical characterization of the aroma of Grenache Rosé wines: Aroma extra dilution analysis, quatitative determination, and sensory reconstitution studies. J. Agric. Food Chem..

[41] Fazzalari F.A. (1978). Compilation of Odor and Taste Threshold Values Data.

[42] Porat R., Deterre S., Giampaoli P., Plotto A., Havkin-Frenkel D., Dudai N. (2016). Chapter 1: The flavour of citrus fruits. Biotechnology in Flavour Production.

[43] Ilc T., Werk-Reichhart D., Navrot N. (2016). Meta-analysis of the core aroma components of grape and wine aroma. Front. Plant Sci..

[44] Black C.A., Parker M., Siebert T.E., Capone D.L., Francis I.L. (2015). Terpenoids and their role in wine flavour: Recent advances. Aust. J. Grape Wine Res..

[45] Oliveira J.M., Araújo I.M., Pereira O.M., Maia J.S., Amaral A.J., Maia M.O. (2004). Characterization and differentiation of five “*Vinhos Verdes*” grape varieties on the basis of monoterpenic compounds. Anal. Chim. Acta.

[46] Burdock G.A. (2010). Fenaroli´s Handbook of Flavour Ingredients.

[47] Li Z., Howell K., Fang Z., Zhang P. (2019). Sesquiterpenes in grapes and wines: Occurrence, biosynthesis, functionality, and influence of winemaking process. Compr. Rev. Food Sci. Food Saf..

[48] Rienth M., Vigneron N., Darriet P., Sweetman C., Burbidge C., Bongui C., Walker R.P., Famiani F., Castellarin S.D. (2021). Grape berry sencondary metabolites and their modulation by abiotic factors in a climate change scenario—A review. Front. Plant Sci..

[49] Petronilho S., Coimbra M.A., Rocha S.M. (2014). A critical review on extraction techniques and gas chromatography-based determination of grapevine derived sesquiterpenes. Anal. Chim. Acta.

[50] Coelho E., Rocha S.M., Delgadillo I., Coimbra M.A. (2006). Headspace-SPME applied to varietal volatile components evolution during *Vitis vinifera* L. cv. ‘Baga’ ripening. Anal. Chim. Acta.

[51] Perestrelo R., Barros A.S., Rocha S.M., Câmara J.S. (2011). Optimisation of solid-phase microextraction combined with gas chromatography-mass spectrometry-based methodology to establish the global volatile signatura in pulp and skin of *Vitis vinifera* L. grape varieties. Talanta.

[52] González-Barreiro C., Rial-Otero R., Cancho-Grande B., Simal-Gándara J. (2015). Wine aroma compounds in grapes: A critical review. Crit. Rev. Food Sci. Nutr..

[53] Canosa P., Oliveira J.M., Masa A., Vilanova M. (2011). Study of the volatile and glycosidically bound compounds of minority *Vitis vinifera* red cultivars from NW Spain. J. Inst. Brew..

[54] Cedrón-Fernández M.T. (2004). Estudio analítico de Compuestos Volátiles En Vino. Caracterización Quimiométrica de Distintas Denominaciones de Origen. Doctoral Dissertation.

[55] Gómez-Plaza E., Gil-Muñoz R., Carreño-Espín J., Fernández-López J.A., Martínez-Cutillas A. (1999). Investigation on the aroma of wines from seven clones of Monastrell grapes. Eur. Food Res. Technol..

[56] Noguerol-Pato R., González-Barreiro C., Cancho-Grande B., Santiago J.L., Martínez M.C., Simal-Gándara J. (2012). Aroma potential of Brancellao grapes from different cluster positions. Food Chem..

[57] López-Tamames E., Carro-Mariño N., Gunata Y.Z., Sapis C., Baumes R., Bayonove C. (1997). Potential aroma in several varieties of Spanish grapes. J. Agric. Food Chem..

[58] Pérez-Navarro J., Da Ros A., Masuero D., Izquierdo-Cañas P.M., Hermosín-Gutiérrez I., Gómez-Alonso S., Mattivi F., Vrhovsek U. (2019). LC-MS/MS analysis of free fatty acid composition and other lipids in skins and seeds of *Vitis vinifera* grape cultivars. Food Res. Int..

[59] Francis I.L., Newton J.L. (2005). Determining wine aroma from compositional data. Aust. J. Grape Wine Res..

[60] Cabaroglu T., Canbas A., Lepoudre J.P., Gunata Z. (2002). Free and bound compound volatile composition of red wines of *Vitis vinifera* L.cv. Öküzgözü and Bogazkere grown in Turkey. Am. J. Enol. Vitic..

[61] Zeller A., Rychlik M. (2007). Impact of estragole and other odorants on the flavour of anise and tarragon. Flavour Fragr. J..

[62] Caven-Quantrill D.J., Buglass A.J. (2007). Determination of volatile organic compounds in English vineyard grape juices by immersion stir bar sorptive extraction-gas chromatography/mass spectrometry. Flavour Fragr. J..

[63] Ruíz-Bejarano M.J., Castro-Mejías R., Rodríguez-Dodero M.D.C., García-Barroso C. (2016). Volatile composition of Pedro Ximénez and muscat sweet sherry wines from sun and chamber dried grapes: A feasible alternative to the traditional sun-drying. J. Food Sci. Technol..

[64] Culbert J.A., Jiang W., Ristic R., Puglisi C.J., Nixon E.C., Shi H., Wilkinson K.L. (2021). Glycosylation of Volatile Phenols in Grapes following Pre-Harvest (On-Vine) vs. Post-Harvest (Off-Vine) Exposure to Smoke. Molecules.

[65] Maturano Y.P., Nally M.C., Assof M.V., Toro M.E., Castellanos de Figueroa L.I., Jofré V.P., Vazquez F. (2018). Free volatile compounds of cv. Pedro Giménez (*Vitis vinifera* L.) white grape must grown in San Juan, Argentina. S. Afr. J. Enol. Vitic..

[66] Furdíková K., Bajnociová L., Malík F., Špánik I. (2017). Investigation of volatile profile of varietal Gewürztraminer wines using two-dimensional gas chromatography. J. Food Nutr. Res..

[67] Dufossé L., Latrasse A., Spinnler H.E. (1994). Importance des lactones dans les arômes alimentairs: Structure, distribution, propiétés sensorielles et biosynthèse. Sci. Aliments.

[68] Roland A., Schenider R., Razungles A., Cavelier F. (2011). Varietal thiols in wine: Discovery, analysis and applications. Chem. Rev..

[69] Lin J., Massonnet M., Cantu D. (2019). The genetic basis of grape and wine aroma. Hortic. Res..

[70] Dubourdieu D., Tominaga T., Moreno-Arribas M.V., Polo M.C. (2009). Polyfunctional Thiol Compounds. Wine Chemistry and Biochemistry.

[71] Liu J., Zhu X.L., Ullah N., Tao Y.S. (2017). Aroma glycosides in grapes and wine. J. Food Sci..

[72] Rodríguez-Bencomo J.J., Cabrera-Valido H.M., Pérez-Trujillo J.P., Cacho J. (2011). Bound aroma compounds of Gual and Listán blanco grape varieties and their influence in the elaborated wines. Food Chem..

[73] Gunata Y.Z., Bayonove C.L., Baumes R.L., Cordonnier R.E. (1985). The aroma of grapes I. Extraction and determination of free and glycosidically bound fractions of some grape aroma components. J. Chromatogr..

[74] Mateo J.J., Jiménez M. (2000). Review: Monoterpenes in grape juice and wines. J. Chromatogr. A.

[75] Welke J.E., Manfroi V., Zanus M., Lazarotto M., Zini C.A. (2012). Characterization of the volatile profile of Brazilian Merlot wines through comprehensive two-dimensional gas chromatography time-of-flight mass spectrometric detection. J. Chromatogr. A.

[76] Rocha S.M., Rodrigues F., Coutinho P., Delgadillo I., Coimbra M.A. (2004). Volatile composition of Baga red wine. Assessment of the identification of the would-be impact odourants. Anal. Chim. Acta.

[77] Ferreira V., Lopez R., Aznar M., Jackson J.F., Linskens H.F. (2002). Olfactometry and aroma extract dilution analysis of wines. Molecular Methods of Plant Analysis. Analysis of Taste and Aroma.

[78] Franco M., Peinado R.A., Medina M., Moreno J. (2004). Off-vine grape drying effect on volatile compounds and aromatic series in must from Pedro Ximénez grape variety. J. Agric. Food Chem..

[79] Genovese A., Lisanti M.T., Gambuti A., Piombino P., Moio L. (2007). Relationship between sensory perception and aroma compounds of monovarietal red wines. Acta Hortic..

[80] Peinado R.A., Moreno J., Bueno J.E., Moreno J.A., Mauricio J.C. (2004). Comparative study of aromatic compounds in two young white wines subjected to prefermentative cryomaceration. Food Chem..

[81] Peinado R.A., Mauricio J.C., Moreno J. (2006). Aromatic series in sherry wines with gluconic acid subjected to different biological aging conditions by *Saccharomyces cerevisiae* var. *capensis*. Food Chem..

[82] García-Carpintero E.G., Sanchez-Palomo E., Gomez Gallego M.A., Gonzalez-Vinas M.A. (2011). Volatile and sensory characterization of red wines from cv. Moravia Agria minority grape variety cultivated in La Mancha region over five consecutive vintages. Food Res. Int..

[83] Latrasse A., Maarse H. (1991). Fruits III. Volatile Compounds in Food and Beverages.

[84] Ubeda C., Cortiella M.G., Galán R.B., Peña-Neira A. (2017). Influence of maturity and vineyard location on free and bound aroma compounds of grapes from the País cultivar. S. Afr. J. Enol. Vitic..

[85] Poitou X., Redon P., Pons A., Bruez E., Delière L., Marchal A., Cholet C., Geny-Denis L., Darriet P. (2021). Methyl salicylate, a grape and wine chemical marker and sensory contributor in wines elaborated from grapes affected or not by cryptogrammic diseases. Food Chem..

[86] Chalal M., Winkler J.B., Gourrat K., Trouvelot S., Adrian M., Schnitzler J.P., Jamois F., Daire X. (2015). Sesquiterpene volatile organic compounds (VOCs) are markers of elicitation by sulphated laminarine in grapevine. Front. Plant Sci..

[87] Korenika A.M.J., Preiner D., Tomaz I., Jeromel A. (2020). Volatile profile characterization of Croatian commercial sparkling wines. Molecules.

[88] Dimitrovska M., Bocevska M., Dimitrovski D., Murkovic M. (2011). Anthocyanin composition of Vranec, Cabernet Sauvignon, Merlot and Pinot Noir grapes as indicator of their varietal differentiation. Eur. Food Res. Technol..

[89] Mattivi F., Guzzon F., Vrhowsek U., Stefanini M., Velasco R. (2006). Metabolite profiling of grape: Flavonols and anthocyanins. J. Agric. Food Chem..

[90] Pomar F., Novo M., Masa A. (2005). Varietal differences among the anthocyanin profiles of 50 red table grapes cultivars studied by high performance liquid chromatography. J. Chromatogr. A.

