# Supplementary material for: Approach to the Chemotaxonomic Characterization of Traditional Cultivation Grape Varieties through Their Varietal Aroma Profile
Source: Foods. 2022 May 15;11(10):1427. doi: 10.3390/foods11101427 (PMC9140530; doi:10.3390/foods11101427)
Supplement: Supplementary file 1 [file foods-11-01427-s001.zip › foods-1668516-supplementary.pdf]

Table S1: Yearly (2015, 2016 and 2017) bioclimatic indices and climatic data in the experimental plot site in north-west Spain (EVEGA, Ourense).

Index	2015		2016		2017	
	Value	Viticulture climate	Value	Viticulture climate	Value	Viticulture climate
HI: Heliothermal	2664.15	Warm (HI+2)	2522.11	Warm (HI+2)	2678.62	Warm (HI+2)
CI: Night cold (ºC)	12.24	Cool nights (CI+1)	11.74	Very cool nights (CI+2)	11.13	Very cool nights (CI+2)
**Parameter**	**Seasonal**	**Annual**	**Seasonal**	**Annual**	**Seasonal**	**Annual**
Max Tª (ºC)	29.31	23.32	28.85	22.81	29.81	23.87
Mean Tª (ºC)	18.92	14.26	18.39	13.92	19.05	14.03
Min Tª (ºC)	10.30	7.23	9.97	7.23	10.35	6.81
Rainfall (mm)	231.20	659.20	382.40	1211.80	228.60	869.00
Days with mean T > 35 ºC	29	29	46	46	28	29

Table S2: White varieties free aromatic fraction profile (Values are expressed as percentages).

Varitey	Year	% ACIDS	% ALCOHOLS	%ALDEHYDES	%C6	%ESTERS	%PHENOLS	%THIOLS
AL	**2015**	22.48	16.64	1.01	40.11	n.d.	0.22	n.d.
	**2016**	9.81	25.55	2.52	45.79	n.d.	0.24	0.29
	**2017**	1.33	20.56	0.87	52.73	n.d.	n.d.	n.d.
		**11.21 ± 10.65 a**	**20.92 ± 4.46 a**	**1.47 ± 0.91 a**	**46.21 ± 6.32 ab**	**n.d. a**	**0.15 ± 0.13 a**	**0.10 ± 0.17 ab**
BB	**2015**	4.51	33.69	8.22	40.90	n.d.	n.d.	0.16
	**2016**	3.15	16.72	0.37	74.12	0.32	0.21	n.d.
		**3.83 ± 0.96 a**	**25.20 ± 12.00 a**	**4.29 ± 5.55 ab**	**57.51 ± 23.48 ab**	**0.16 ± 0.22 a**	**0.11 ± 0.15 a**	**0.08 ± 0.12 ab**
CBL	**2015**	15.52	17.31	3.48	44.37	n.d.	0.63	n.d.
	**2016**	11.07	39.78	1.34	23.73	10.08	0.25	n.d.
	**2017**	13.45	21.14	1.16	53.29	n.d.	2.11	n.d.
		**13.35 ± 2.23 a**	**26.08 ± 12.02 a**	**1.99 ± 1.29 ab**	**40.46 ± 15.16 ab**	**3.36 ± 5.82 a**	**1.00 ± 0.98 a**	**n.d. ab**
LO	**2015**	11.89	29.25	1.30	37.08	8.00	n.d.	n.d.
	**2016**	6.17	12.78	0.45	48.72	n.d.	n.d.	n.d.
	**2017**	2.28	9.72	0.42	37.08	n.d.	0.48	n.d.
		**6.78 ± 4.83 a**	**17.25 ± 10.50 a**	**0.72 ± 0.50 a**	**40.96 ± 6.72 ab**	**2.67 ± 4.62 a**	**0.16 ± 0.28 a**	**n.d. ab**
VB	**2015**	23.61	21.09	2.70	48.05	n.d.	0.42	n.d.
	**2016**	14.88	15.20	1.21	55.61	0.32	0.18	n.d.
	**2017**	15.14	14.59	0.99	41.55	n.d.	n.d.	n.d.
		**17.88 ± 4.96 a**	**16.96 ± 3.59 a**	**1.63 ± 0.93 a**	**48.40 ± 7.03 ab**	**0.11 ± 0.19 a**	**0.20 ± 0.21 a**	**n.d. a**
**Significance**		**ns**	**ns**	**ns**	**ns**	**ns**	**ns**	**ns**

Varitey	Year	%KETONES	%PAH´s	%LACTONS	%NORISOPRENOIDS	%SESQUITERPENES	%TERPENES
AL	**2015**	2.32	1.27	n.d.	1.42	n.d.	14.53
	**2016**	4.60	3.50	1.17	n.d.	n.d.	6.53
	**2017**	2.67	2.99	n.d.	n.d.	n.d.	18.85
		**3.20 ± 1.23 abc**	**2.58 ± 1.17 ab**	**0.39 ± 0.67 ab**	**0.47 ± 0.82 a**	**n.d. a**	**13.31 ± 6.25 b**
BB	**2015**	1.95	4.09	0.98	n.d.	n.d.	5.51
	**2016**	1.14	1.03	0.58	0.42	n.d.	1.95
		**1.54 ± 0.57 ab**	**2.56 ± 2.16 ab**	**0.78 ± 0.28 abc**	**0.21 ± 0.30 a**	**n.d. a**	**3.73 ± 2.52 ab**
CBL	**2015**	7.76	4.90	1.63	2.33	n.d.	2.07
	**2016**	4.50	5.04	2.41	0.32	n.d.	1.49
	**2017**	2.62	1.15	1.42	1.69	n.d.	1.98
		**4.96 ± 2.60 c**	**3.70 ± 2.21 b**	**1.82 ± 0.52 c**	**1.45 ± 1.03 b**	**n.d. a**	**1.84 ± 0.31 ab**
LO	**2015**	0.51	0.55	0.06	0.50	n.d.	10.86
	**2016**	3.60	2.68	2.34	n.d.	n.d.	23.26
	**2017**	1.15	1.17	0.51	0.15	n.d.	47.05
		**1.75 ± 1.63 ab**	**1.47 ± 1.09 a**	**0.97 ± 1.21 abc**	**0.21 ± 0.26 a**	**n.d. a**	**27.06 ± 18.39 c**
VB	**2015**	0.88	0.45	n.d.	0.03	n.d.	2.77
	**2016**	0.80	1.78	0.09	0.24	n.d.	9.68
	**2017**	2.64	1.65	n.d.	0.93	n.d.	22.52
		**1.44 ± 1.04 a**	**1.29 ± 0.73 a**	**0.03 ± 0.05 a**	**0.40 ± 0.47 a**	**n.d. a**	**11.66 ± 10.02 ab**
**Significance**		**ns**	**ns**	**ns**	**ns**	**ns**	**

PAH´s: Polycyclic Aromatic Hidrocarbons; AL: 'Albariño'; BB: 'Brancellao Blanco'; CBL: 'Caíño Blanco'; LO: 'Loureira'; VB: 'Verdello Blanco'; n.d.: no detected. ** and ns indicate significance at P≤0.01 and not

significant difference respectively. Mean value, SD and different roman letters (a-c), showing significant differences according to Fisher's test (P<0.05), are indicated in bold for each variety.

Table S3: Red varieties free aromatic fraction profile (Values are expressed as percentages).

Varitey	Year	% ACIDS	% ALCOHOLS	%ALDEHYDES	%C6	%ESTERS	%PHENOLS	%THIOLS
BR	**2015**	15.06	13.74	9.51	58.81	0.21	n.d.	n.d.
	**2016**	3.56	14.43	3.60	72.88	0.29	n.d.	n.d.
	**2017**	6.32	16.49	1.90	61.61	n.d.	1.96	n.d.
		**8.31 ± 6.00 a**	**14.89 ± 1.43 a**	**5.01 ± 3.99 b**	**64.44 ± 7.45 b**	**0.17 ± 0.15 a**	**0.65 ± 1.13 a**	**n.d. a**
CB	**2015**	25.14	22.16	1.92	40.62	n.d.	0.43	n.d.
	**2016**	12.70	21.18	0.39	54.99	0.25	0.31	n.d.
	**2017**	10.09	28.56	2.39	48.77	n.d.	0.27	n.d.
		**15.98 ± 8.04 a**	**23.97 ± 4.01 a**	**1.57 ± 1.05 a**	**48.13 ± 7.20 ab**	**0.08 ± 0.14 a**	**0.34 ± 0.08 a**	**n.d. a**
CL1	**2015**	29.91	17.93	0.51	40.36	n.d.	0.31	n.d.
	**2016**	0.00	7.98	1.75	85.87	0.19	0.05	n.d.
	**2017**	11.94	24.03	0.00	52.04	0.14	0.53	n.d.
		**13.95 ± 15.06 a**	**16.65 ± 8.10 a**	**0.75 ± 0.90 a**	**59.42 ± 23.64 ab**	**0.11 ± 0.10 a**	**0.30 ± 0.24 a**	**n.d. a**
CL2	**2015**	15.41	16.83	0.62	40.01	n.d.	0.37	n.d.
	**2016**	3.56	13.13	0.93	76.76	0.36	0.13	n.d.
	**2017**	3.15	19.66	0.51	64.29	n.d.	0.93	n.d.
		**7.37 ± 6.96 a**	**16.54 ± 3.27 a**	**0.69 ± 0.22 a**	**60.36 ± 18.69 ab**	**0.12 ± 0.21 a**	**0.48 ± 0.41 a**	**n.d. a**
CT	**2015**	20.13	19.97	2.93	43.99	n.d.	0.39	n.d.
	**2016**	3.14	11.22	3.96	78.11	0.16	n.d.	n.d.
	**2017**	9.69	16.43	0.00	64.70	n.d.	0.90	n.d.
		**10.99 ± 8.57 a**	**15.87 ± 4.40 a**	**2.30 ± 2.06 ab**	**62.26 ± 17.19 ab**	**0.05 ± 0.09 a**	**0.43 ± 0.45 a**	**n.d. a**
CS	**2015**	19.45	13.04	0.94	51.17	n.d.	0.46	1.87
	**2016**	11.45	17.17	0.67	62.04	n.d.	0.49	n.d.
	**2017**	8.29	19.43	1.29	60.61	0.17	1.74	n.d.
		**13.06 ± 5.75 a**	**16.55 ± 3.24 a**	**0.97 ± 0.31 a**	**57.94 ± 5.91 ab**	**0.06 ± 0.10 a**	**0.89 ± 0.73 a**	**0.62 ± 1.08 b**
SO	**2016**	4.42	16.29	1.20	72.55	0.97	n.d.	0.13
	**2017**	14.45	27.79	1.06	47.89	n.d.	0.27	n.d.
		**9.43 ± 7.09 a**	**22.04 ± 8.13 a**	**1.13 ± 0.10 a**	**60.22 ± 17.44 ab**	**0.49 ± 0.69 a**	**0.13 ± 0.19 a**	**0.07 ± 0.09 ab**
**Significance**		**ns**	**ns**	**ns**	**ns**	**ns**	**ns**	**ns**

Varitey	Year	%KETONES	%PAH´s	%LACTONS	%NORISOPRENOIDS	%SESQUITERPENES	%TERPENES
BR	**2015**	0.88	0.28	0.24	n.d.	n.d.	1.26
	**2016**	2.05	1.73	1.19	n.d.	n.d.	0.26
	**2017**	3.03	1.68	n.d.	n.d.	n.d.	7.00
		**1.99 ± 1.08 abc**	**1.23 ± 0.82 a**	**0.48 ± 0.63 abc**	n.d. a	n.d. **a**	**2.84 ± 3.64 ab**
CB	**2015**	4.44	2.27	1.28	0.46	n.d.	1.27
	**2016**	4.70	3.38	1.69	n.d.	n.d.	0.40
	**2017**	4.87	3.23	0.27	0.10	n.d.	1.43
		**4.67 ± 0.22 bc**	**2.96 ± 0.61 ab**	**1.08 ± 0.73 abc**	**0.19 ± 0.24 a**	**n.d. a**	**1.04 ± 0.56 ab**
CL1	**2015**	5.10	2.16	1.61	0.72	n.d.	1.40
	**2016**	1.75	0.85	1.32	n.d.	n.d.	0.24
	**2017**	4.51	2.03	0.19	0.59	n.d.	3.99
		**3.79 ± 1.79 abc**	**1.68 ± 0.72 ab**	**1.04 ± 0.75 abc**	**0.44 ± 0.38 a**	**n.d. a**	**1.88 ± 1.92 ab**
CL2	**2015**	5.64	3.11	0.18	1.66	n.d.	16.16
	**2016**	2.16	1.17	0.45	n.d.	n.d.	1.34
	**2017**	4.31	0.86	3.42	n.d.	n.d.	2.87

		**4.04 ± 1.76 abc**	**1.71 ± 1.22 ab**	**1.35 ± 1.80 abc**	**0.55 ± 0.96 ab**	n.d. **a**	**6.79 ± 8.15 ab**
**CT**	**2015**	7.11	2.51	1.45	1.25	n.d.	0.27
	**2016**	1.04	1.09	1.10	n.d.	n.d.	0.17
	**2017**	3.69	2.10	1.15	0.49	0.05	0.79
		**3.95 ± 3.04 abc**	**1.90 ± 0.73 ab**	**1.23 ± 0.19 abc**	**0.58 ± 0.63 ab**	**0.02 0.03 b**	**0.41 ± 0.33 a**
**CS**	**2015**	6.15	5.12	1.48	0.10	n.d.	0.21
	**2016**	4.29	2.05	1.60	n.d.	n.d.	0.24
	**2017**	3.68	2.01	2.06	0.24	n.d.	0.48
		**4.71 ± 1.29 bc**	**3.06 ± 1.79 b**	**1.71 ± 0.30 bc**	**0.12 ± 0.12 a**	n.d. **a**	**0.31 ± 0.15 a**
**SO**	**2016**	2.06	1.32	0.76	n.d.	n.d.	0.30
	**2017**	5.92	1.30	0.94	0.21	n.d.	0.18
		**3.99 ± 2.73 abc**	**1.31 ± 0.01 a**	**0.85 ± 0.13 abc**	**0.10 ± 0.15 a**	n.d. **a**	**0.24 ± 0.09 a**
**Significance**		**ns**	**ns**	**ns**	**ns**	**ns**	**

PAH´s: Polycyclic Aromatic Hidrocarbons; BR: 'Brancellao'; CB: 'Caíño Bravo'; CL1: 'Caíño Longo 1'; CL2: 'Caíño Longo 2'; CT: 'Caíño Tinto'; CS: 'Castañal'; SO: 'Sousón'. n.d.: no detected. ** and ns indicate significance at P≤0.01 and not significant difference respectively. Mean value, SD and different roman letters (a-c), showing significant differences according to Fisher's test (P<0.05), are indicated in bold for each variety.

Table S4: White varieties aromatic precursor fraction profile (Values are expressed as percentages).

Varitey	Year	% ACIDS	% ALCOHOLS	%ALDEHYDES	%C6	%ESTERS	%PHENOLS	%THIOLS
AL	2015	10.90	35.71	n.d.	4.83	10.95	4.97	n.d.
	2016	2.40	11.46	n.d.	1.96	36.12	41.98	n.d.
	2017	13.75	26.47	n.d.	2.72	31.37	2.11	0.11
		**9.02 ± 5.90 b**	**24.55 ± 12.24 a**	**n.d. a**	**3.17 ± 1.49 a**	**26.15 ± 13.37 b**	**16.36 ± 22.24 abcd**	**0.04 ± 0.07 a**
BB	2015	0.00	54.40	n.d.	14.36	1.48	15.71	0.48
	2016	0.98	28.21	n.d.	5.83	6.76	54.82	0.21
		**0.49 ± 0.69 ab**	**41.30 ± 18.51 abcd**	**n.d. a**	**10.10 ± 6.03 abc**	**4.12 ± 3.73 a**	**35.27 ± 27.65 cd**	**0.35 ± 0.19 ab**
CBL	2015	10.07	48.69	1.66	6.96	15.88	8.66	0.22
	2016	2.92	72.97	2.39	5.00	8.93	4.44	n.d.
	2017	0.82	65.32	2.78	3.91	12.79	2.86	n.d.
		**4.60 ± 4.85 ab**	**62.33 ± 12.41 d**	**2.28 ± 0.57 abc**	**5.29 ± 1.55 ab**	**12.53 ± 3.48 ab**	**5.32 ± 3.00 ab**	**0.07 ± 0.13 a**
LO	2015	2.87	34.33	n.d.	3.16	5.14	0.89	0.24
	2016	18.39	43.51	2.88	2.31	20.36	8.91	n.d.
	2017	0.96	59.88	n.d.	3.09	3.45	4.75	0.10
		**7.41 ± 9.56 ab**	**45.91 ± 12.95 abcd**	**0.96 ± 1.66 abc**	**2.85 ± 0.47 a**	**9.65 ± 9.31 ab**	**4.85 ± 4.01 ab**	**0.11 ± 0.12 ab**
VB	2015	0.52	35.41	n.d.	4.26	1.90	22.86	n.d.
	2016	1.74	35.51	n.d.	9.89	9.75	28.65	n.d.
	2017	2.37	30.25	n.d.	4.07	6.62	32.15	n.d.
		**1.55 ± 0.94 ab**	**33.72 ± 3.01 abc**	**n.d. a**	**6.07 ± 3.30 ab**	**6.09 ± 3.95 a**	**27.89 ± 4.69 bcd**	**n.d. a**
**Significance**		**ns**	*****	**ns**	*******	**ns**	*****	**ns**

Varitey	Year	%KETONES	%PAH´s	%LACTONS	%NORISOPRENOIDS	%SESQUITERPENES	%TERPENES
AL	2015	0.43	0.12	n.d.	0.23	n.d.	31.85
	2016	0.41	0.07	n.d.	n.d.	0.01	5.58
	2017	0.48	0.43	n.d.	n.d.	n.d.	22.55
		**0.44 ± 0.04 a**	**0.21 ± 0.19 ab**	**n.d. a**	**0.08 ± 0.14 a**	**n.d. a**	**19.99 ± 13.32 ab**
BB	2015	0.48	0.23	n.d.	n.d.	0.02	12.83
	2016	0.32	0.05	n.d.	n.d.	0.01	2.80
		**0.40 ± 0.11 a**	**0.14 ± 0.13 ab**	**n.d. a**	**n.d. a**	**0.02 ± 0.01 ab**	**7.82 ± 7.09 ab**
CBL	2015	0.64	0.05	n.d.	n.d.	0.04	7.11
	2016	1.26	0.28	n.d.	n.d.	n.d.	1.82
	2017	0.74	0.35	n.d.	0.07	n.d.	10.36
		**0.88 ± 0.33 a**	**0.22 ± 0.16 ab**	**n.d. a**	**0.02 ± 0.04 a**	**0.01 ± 0.02 a**	**6.43 ± 4.31 ab**
LO	2015	0.10	0.02	n.d.	n.d.	n.d.	53.26
	2016	2.32	0.56	n.d.	n.d.	n.d.	0.76
	2017	0.58	0.44	n.d.	0.08	n.d.	26.67
		**1.00 ± 1.17 a**	**0.34 ± 0.28 ab**	**n.d. a**	**0.03 ± 0.05 a**	**n.d. a**	**26.90 ± 26.25 b**
VB	2015	0.05	0.06	n.d.	n.d.	0.04	34.89
	2016	0.32	0.11	n.d.	n.d.	0.03	14.01
	2017	0.55	0.21	n.d.	n.d.	0.05	23.72
		**0.31 ± 0.25 a**	**0.13 ± 0.08 ab**	**n.d. a**	**n.d. a**	**0.04 ± 0.01 b**	**24.20 ± 10.45 ab**
**Significance**		**ns**	**ns**	**ns**	**ns**	*****	**ns**

PAH´s: Polycyclic Aromatic Hidrocarbons; AL: 'Albariño'; BB: 'Brancellao Blanco'; CBL: 'Caíño Blanco'; LO: 'Loureira'; VB: 'Verdello Blanco'; n.d.: no detected. *, *** and ns indicate significance at P≤0.05, P≤0.001

and not significant difference respectively. Mean value, SD and different roman letters (a-d), showing significant differences according to Fisher's test ($P<0.05$), are indicated in bold for each variety.

Table S5: Red varieties aromatic precursor fraction profile (Values are expressed as percentages).

Varitey	Year	% ACIDS	% ALCOHOLS	%ALDEHYDES	%C6	%ESTERS	%PHENOLS	%THIOLS
BR	**2016**	0.40	58.28	6.95	21.35	6.23	2.89	n.d.
	**2017**	3.35	44.72	0.50	13.07	17.42	3.22	0.26
		**1.87 ± 2.08 ab**	**51.50 ± 9.59 bcd**	**3.73 ± 4.56 bc**	**17.21 ± 5.85 cd**	**11.83 ± 7.92 ab**	**3.05 ± 0.24 ab**	**0.13 ± 0.19 ab**
CB	**2015**	n.d.	34.06	1.49	16.39	3.97	0.58	0.08
	**2016**	n.d.	58.69	9.48	12.71	7.24	6.13	n.d.
	**2017**	0.12	45.54	4.25	7.68	30.06	2.93	0.51
		**0.04 ± 0.07 ab**	**46.10 ± 12.33 abcd**	**5.08 ± 4.06 c**	**12.26 ± 4.38 bc**	**13.75 ± 14.21 ab**	**3.22 ± 2.79 ab**	**0.20 ± 0.28 ab**
CL1	**2015**	0.21	53.77	0.12	23.28	17.89	1.74	n.d.
	**2016**	9.97	57.33	3.19	10.53	11.81	4.12	n.d.
	**2017**	2.26	46.97	1.51	9.84	25.49	1.98	0.27
		**4.15 ± 5.15 ab**	**52.69 ± 5.27 cd**	**1.61 ± 1.54 abc**	**14.55 ± 7.56 cd**	**18.40 ± 6.86 ab**	**2.61 ± 1.31 a**	**0.09 ± 0.16 a**
CL2	**2015**	1.43	60.68	2.83	6.93	7.93	1.02	0.08
	**2016**	0.89	8.80	0.80	1.24	37.66	46.28	n.d.
	**2017**	3.44	16.39	n.d.	1.67	4.65	63.57	n.d.
		**1.92 ± 1.34 ab**	**28.62 ± 28.02 ab**	**1.21 ± 1.46 ab**	**3.28 ± 3.17 a**	**16.74 ± 18.19 ab**	**36.96 ± 32.30 d**	**0.03 ± 0.04 a**
CT	**2015**	2.00	58.76	2.10	22.74	11.02	1.01	0.05
	**2016**	n.d.	66.93	5.57	15.09	5.00	1.85	n.d.
	**2017**	3.13	59.44	2.92	11.05	15.71	1.34	0.35
		**1.71 ± 1.59 ab**	**61.71 ± 4.53 d**	**3.53 ± 1.81 bc**	**16.29 ± 5.93 cd**	**10.58 ± 5.37 ab**	**1.40 ± 0.42 a**	**0.14 ± 0.19 ab**
CS	**2015**	1.07	47.46	2.29	20.81	0.80	18.96	0.05
	**2016**	11.82	58.50	2.50	20.60	1.36	4.14	n.d.
	**2017**	1.61	48.58	2.93	17.31	5.34	11.73	0.29
		**4.83 ± 6.06 ab**	**51.51 ± 6.08 cd**	**2.57 ± 0.33 abc**	**19.57 ± 1.96 d**	**2.50 ± 2.47 a**	**11.61 ± 7.41 abc**	**0.11 ± 0.15 ab**
SO	**2016**	n.d.	47.06	4.08	32.62	4.30	5.78	0.90
	**2017**	0.95	38.13	1.97	29.57	19.91	1.41	0.04
		**0.47 ± 0.67 ab**	**42.59 ± 6.31 abcd**	**3.02 ± 1.49 abc**	**31.09 ± 2.16 e**	**12.10 ± 11.04 ab**	**3.60 ± 3.09 ab**	**0.47 ± 0.61 b**
**Significance**		**ns**	*****	**ns**	*******	**ns**	*****	**ns**

Varitey	Year	%KETONES	%PAH´s	%LACTONS	%NORISOPRENOIDS	%SESQUITERPENES	%TERPENES
BR	**2016**	2.57	0.38	n.d.	n.d.	n.d.	0.96
	**2017**	0.90	0.48	n.d.	n.d.	0.05	16.03
		**1.73 ± 1.18 a**	**0.43 ± 0.07 ab**	**n.d. a**	**n.d. a**	**0.02 ± 0.03 ab**	**8.50 ± 10.65 ab**
CB	**2015**	0.52	n.d.	n.d.	n.d.	n.d.	42.91
	**2016**	4.25	0.84	n.d.	n.d.	n.d.	0.66
	**2017**	0.50	0.25	n.d.	n.d.	n.d.	8.16
		**1.75 ± 2.16 a**	**0.36 ± 0.43 ab**	**n.d. a**	**n.d. a**	**n.d. ab**	**17.24 ± 22.54 ab**
CL1	**2015**	0.05	n.d.	n.d.	0.19	n.d.	2.76
	**2016**	2.16	0.34	n.d.	n.d.	n.d.	0.54
	**2017**	0.74	0.30	n.d.	n.d.	n.d.	10.64
		**0.98 ± 1.08 a**	**0.22 ± 0.19 ab**	**n.d. a**	**0.06 ± 0.11 a**	**n.d. a**	**4.64 ± 5.31 a**
CL2	**2015**	0.07	n.d.	0.03	0.09	n.d.	18.91
	**2016**	0.25	0.02	n.d.	n.d.	n.d.	4.06
	**2017**	0.32	n.d.	n.d.	n.d.	0.04	9.92
		**0.22 ± 0.13 a**	**0.01 ± 0.01 a**	**0.01 ± 0.02 b**	**0.03 ± 0.05 a**	**0.01 ± 0.02 a**	**10.97 ± 7.48 ab**
CT	**2015**	0.17	n.d.	n.d.	n.d.	n.d.	2.14

	**2016**	1.69	1.03	n.d.	n.d.	n.d.	2.84
	**2017**	1.25	0.45	n.d.	n.d.	n.d.	4.37
		**1.04 ± 0.78 a**	**0.49 ± 0.52 ab**	**n.d. a**	**n.d. a**	**n.d. a**	**3.12 ± 1.14 a**
**CS**	**2015**	1.07	0.18	n.d.	n.d.	n.d.	7.32
	**2016**	0.40	0.56	n.d.	n.d.	n.d.	0.13
	**2017**	0.91	0.27	n.d.	n.d.	n.d.	11.02
		**0.79 ± 0.35 a**	**0.34 ± 0.20 ab**	**n.d. a**	**n.d. a**	**n.d. a**	**6.16 ± 5.54 ab**
**SO**	**2016**	1.45	1.09	n.d.	n.d.	n.d.	2.74
	**2017**	0.55	0.24	n.d.	n.d.	n.d.	7.23
		**1.00 ± 0.63 a**	**0.67 ± 0.60 b**	**n.d. a**	**n.d. a**	**n.d. a**	**4.98 ± 3.18 ab**
**Significance**		**ns**	**ns**	**ns**	**ns**	*****	**ns**

PAH´s: Polycyclic Aromatic Hidrocarbons; BR: 'Brancellao'; CB: 'Caíño Bravo'; CL1: 'Caíño Longo 1'; CL2: 'Caíño Longo 2'; CT: 'Caíño Tinto'; CS: 'Castañal'; SO: 'Sousón'. n.d.: no detected. *, *** and ns indicate significance at P≤0.05, P≤0.001 and not significant difference respectively. Mean value, SD and different roman letters (a-e), showing significant differences according to Fisher's test (P<0.05), are indicated in bold for each variety.
